# Fully forward mode training for optical neural networks

**DOI:** 10.1038/s41586-024-07687-4

**Published:** 2024-08-07

**Authors:** Zhiwei Xue, Tiankuang Zhou, Zhihao Xu, Shaoliang Yu, Qionghai Dai, Lu Fang

**Affiliations:** 1 Department of Electronic Engineering, Tsinghua University, Beijing, China; 2 Beijing National Research Center for Information Science and Technology, Tsinghua University, Beijing, China; 3 Institute for Brain and Cognitive Sciences, Tsinghua University, Beijing, China; 4 Shenzhen International Graduate School, Tsinghua University, Shenzhen, China; 5 Research Center for Intelligent Optoelectronic Computing, Zhejiang Laboratory, Hangzhou, China; 6 Department of Automation, Tsinghua University, Beijing, China

**Keywords:** Computer science, Electrical and electronic engineering

## Abstract

Optical computing promises to improve the speed and energy efficiency of machine learning applications^1–6^. However, current approaches to efficiently train these models are limited by in silico emulation on digital computers. Here we develop a method called fully forward mode (FFM) learning, which implements the compute-intensive training process on the physical system. The majority of the machine learning operations are thus efficiently conducted in parallel on site, alleviating numerical modelling constraints. In free-space and integrated photonics, we experimentally demonstrate optical systems with state-of-the-art performances for a given network size. FFM learning shows training the deepest optical neural networks with millions of parameters achieves accuracy equivalent to the ideal model. It supports all-optical focusing through scattering media with a resolution of the diffraction limit; it can also image in parallel the objects hidden outside the direct line of sight at over a kilohertz frame rate and can conduct all-optical processing with light intensity as weak as subphoton per pixel (5.40 × 10^18^- operations-per-second-per-watt energy efficiency) at room temperature. Furthermore, we prove that FFM learning can automatically search non-Hermitian exceptional points without an analytical model. FFM learning not only facilitates orders-of-magnitude-faster learning processes, but can also advance applied and theoretical fields such as deep neural networks, ultrasensitive perception and topological photonics.

## Abstract (web summary)

We present fully forward mode learning, which conducts machine learning operations on site, leading to faster learning and promoting advancement in numerous fields.

## Main

**Fig. 1:**
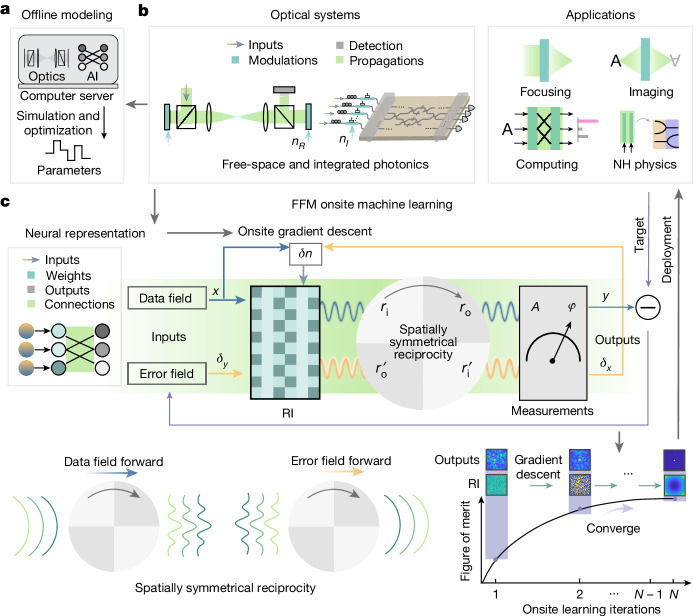
FFM onsite machine learning for optical systems. **a**, Conventionally, optics-related AI is designed through offline modelling and optimization, leading to limited design efficiency and system performance. **b**, General optical systems, including free-space lens optics and integrated photonics, contain the modulation regions (dark green) and propagation regions (light green), where the refractive indexes are respectively tunable and fixed. **c**, These regions in the optical system can be mapped to weights and neuron connections in the neural representation, which enables the construction of a differentiable onsite neural network between the input and output (top-left panel). With spatially symmetrical reciprocity, the data and error computes share forward physical propagations and measurements, and the onsite gradients are calculated for the update of the refractive indexes in the design region (top-right and bottom-left panels). Through onsite gradient descent, the optical system gradually converges (bottom-right panel). RI, refractive index; A, amplitude; Φ, phase.

The rapid advancement of machine learning is transforming the sciences and technology^1–3,7–9^. Taking advantage of optics and photonics, optical artificial intelligence (AI) can realize large-bandwidth and high-energy-efficiency computing^3–6,10^. Simultaneously, the utilization of AI for optics can improve the design and control of optical systems^11–16^. Nonetheless, both optical AI and AI for optics are implemented in silico on electronic computers and thus require strict modelling and large amounts of training data to extract the system information (Fig. 1a). Optical AI methods primarily involve optical emulation of electronic artificial neural networks (ANNs), with the photonic architecture design being executed on electronic computers^17–22^. Accordingly, correcting the experimental system error has been a challenge requiring extensive effort to characterize the optical propagation spatially and temporally^18,23,24^. In AI for optical design^12,25–27^, the system must also be modelled analytically or implicitly. As the system complexity increases, both analytical and numerical modelling become time-consuming, and the required computational resources grow exponentially. More importantly, because of system imperfections and the complexity of light-wave propagation, perfectly precise modelling of a general optical system is impossible, and a mismatch always occurs between the offline model and the real system.

To address the challenges associated with offline modelling, it would be ideal to implement the machine learning algorithms on site^28–31^. However, achieving high accuracy and high efficiency in parallel onsite learning of large-scale systems remains challenging. The efficiency of perturbation-based optimization methods is limited^32,33^. More efficient gradient-based methods are typically confined to small-scale and simple problems because the requirement of backward-propagating light fields necessitates stringent system calibration and alignment^34–36^. Hybrid methods have also been explored that combine onsite forward propagation with offline backward propagation using electronic computers to implement the gradient descent algorithm^24,37,38^. Nonetheless, the offline modelling that remains necessary in the backward propagation processes may not always be achievable in generic optical systems.

The technical challenges inhibiting the realization of model-free and onsite machine learning in optical systems include the following. First, a unified differentiable onsite parametric formulation is greatly needed for general optics. A conflict also exists between the system complexity and the learning efficiency/optimality. Existing gradient descent training relies on the back-propagation of the light fields, but the associated complexity makes it difficult to scale up and fully physically implement.

Here we present fully forward mode (FFM) learning for onsite machine learning in free-space and integrated optical systems. Our method maps optical systems to parameterized onsite neural networks, and enables self-learning guided by targets of applications. By leveraging spatial symmetry and Lorentz reciprocity, the necessity of backward propagation in the gradient descent training is eliminated. Consequently, the optical parameters can be self-designed directly on the original physical system. Our approach calculates the gradient based on the measured output light fields from data and error propagations, then efficiently updates the parameters using the gradient descent algorithm. We demonstrate the versatility of the FFM learning method in advancing distinct fields at both the free-space and integrated photonic scales to realize deep optical neural networks (ONNs), high-resolution scattering imaging, dynamic all-optical non-line-of-sight systems, and model-free exceptional point searching in non-Hermitian systems.

## Principles of FFM onsite machine learning

Non-magnetic optical systems are characterized by refractive index, gain and loss, implying that the learning of such an optical system should involve efficient optimization of the complex refractive index $n={n}_{{\rm{R}}}+{\rm{j}}{n}_{{\rm{I}}}$ (*n*_R_, real part of the complex refractive index; *n*_I_, imaginary part of the complex refractive index; j, imaginary unit). According to the tunability of the refractive index, the free-space and integrated optical systems can be divided into two distinct regions: the modulation region and the propagation region^39^, enabling various functionalities (Fig. 1b). We find that an optical system governed by Maxwell’s equations can be reparametrized into a differentiable embedded photonic neural network formulated as

1 $${\bf{y}}={\rm{W}}({\bf{M}}\cdot {\bf{x}}),$$

where **x** and **y** are the input and output electric fields, and **W** and **M** are the propagation and modulation regions characterized by fixed and tunable refractive indexes, respectively (top-left panel of Fig. 1c; proof is given in Supplementary Note 1).

Gradient descent training of neural networks has been central to the renaissance of AI^2,40^. In Fig. 1c, we illustrate FFM gradient descent learning. The light propagation in equation (1) is reformulated as $y({r}_{{\rm{o}}})=\,\int \text{d}({r}_{{\rm{i}}})G({r}_{{\rm{o}}},{r}_{{\rm{i}}})x({r}_{{\rm{i}}})$, where $G({r}_{{\rm{o}}},{r}_{{\rm{i}}})$ is the Green’s function, and *r*_o_ and *r*_i_ designate the output and input indices, respectively. Let *δ*_*y*_ denote the error between the target and output. With Lorentz reciprocity, the error propagation is represented by ${\delta }_{x}({r}_{{\rm{i}}})=\,\int \text{d}({r}_{{\rm{o}}})G({r}_{{\rm{i}}},{r}_{{\rm{o}}}){\delta }_{y}({r}_{{\rm{o}}})$, which corresponds to feeding in the ${\delta }_{y}\left({r}_{{\rm{o}}}\right)$ at the output ports of the system and propagating the field backwards to the input port. To implement gradient descent on an optical system with fully forward propagation, the backward propagation should be replaced by

2 $${\delta }_{x}({r}_{{\rm{o}}}^{{\prime} })=\int \text{d}({r}_{{\rm{i}}}^{{\prime} })\,G({r}_{{\rm{o}}}^{{\prime} },{r}_{{\rm{i}}}^{{\prime} }){\delta }_{y}({r}_{{\rm{i}}}^{{\prime} }).$$

A sufficient condition for the validity of equation (2) is that for every coordinate pair $({r}_{{\rm{i}}},{r}_{{\rm{o}}})$, a pair of $({r}_{{\rm{o}}}^{{\prime} },{r}_{{\rm{i}}}^{{\prime} })$ is always available such that $G({r}_{{\rm{o}}}^{{\prime} },{r}_{{\rm{i}}}^{{\prime} }){\delta }_{y}({r}_{{\rm{i}}}^{{\prime} })=G({r}_{{\rm{i}}},{r}_{{\rm{o}}}){\delta }_{y}({r}_{{\rm{o}}})$, and one-to-one correspondences exist between *r*_i_ and ${r}_{{\rm{o}}}^{{\prime} }$, and between ${r}_{{\rm{i}}}^{{\prime} }$ and *r*_o_. This condition is satisfied in a spatially symmetrical system (bottom-left panel of Fig. 1c), which is inherent in the physical system^41^ (details of derivations are presented in Supplementary Notes 2 and  3). The outputs of the error propagation are then used for calculating the gradients *δn* with respect to the refractive index. After the refractive index in the design region converges, the system is deployed to implement the target applications (details of FFM learning protocols are presented in Supplementary Note 5). The bottom-right panel of Fig. 1c illustrates the design of the free-space system to focus the input light beam. With the application of FFM learning, the figure of merit progressively increases and the designed refractive index profiles gradually approach a parabolic wavefront, leading to the convergence of the output to a tight focal point.

## Parallel FFM gradient descent on deep ONNs

**Fig. 2:**
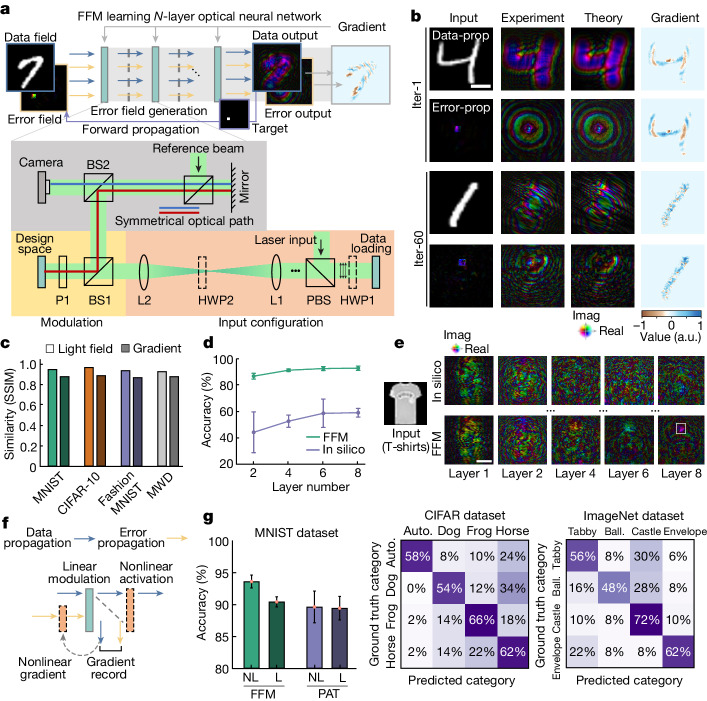
Parallel FFM gradient descent for deep ONNs. **a**, A deep free-space ONN and the experimental system. Dashed grey lines indicate the symmetry plane. **b**, Experimental measurements compared with the analytical simulation. **c**, Structural similarity index between the experimental and simulation light fields. **d**, Experimental accuracies of FFM and in silico designed multilayer ONNs. Error bar delineates twice the standard deviation. **e**, Per-layer outputs of an eight-layer ONN designed with FFM learning compared with in silico optimization. **f**, Incorporation of nonlinearity into FFM learning. **g**, Training nonlinear ONNs on MNIST, CIFAR, and ImageNet datasets with the FFM. Scale bar, 1 mm. a.u., arbitrary unit; auto., automobile; ball., basketball; BS1/BS2, beam splitters; HWP1/HWP2, half-wave plates; L1/L2, lenses; P1, polarizer; PBS, polarized beam splitter; prop, propagation; iter, iteration; NL, nonlinear; L, linear. Source Data

Figure 2a showcases the self-training process of a free-space ONN using FFM learning. To demonstrate the effectiveness of FFM learning, we first trained a one-layer ONN for object classification using benchmark datasets (Supplementary Fig. 1). Figure 2b visualizes the training results on the MNIST dataset. The structural similarity index (SSIM) between the experimental and theoretical light field exceeded 0.97, indicating a high level of similarity (Fig. 2c). It is worth noting that because of the system imperfections, the theoretical results of light fields and gradients do not precisely represent the physical ones, such that the theoretical results are only a reflection of fidelity and therefore should not be regarded as the ground truth. We then investigated multilayer ONNs for classification of the Fashion-MNIST dataset (Fig. 2d). By increasing the number of layers from two to eight, we observed that the experimental testing results for in silico trained networks had average accuracies (twice standard deviations) of 44.0% (35.1%), 52.4% (8.8%), 58.4% (18.4%) and 58.8% (5.5%), respectively. These results were lower than the theoretical accuracies of 92.2%, 93.8%, 96.0% and 96.0%. With FFM learning, the network performances improved to 86.5%, 91.0%, 92.3% and 92.5%, approaching the ideal in silico accuracies. Fig. 2e depicts the outputs of an eight-layer ONN. As the layer number increases, the experimental outputs of in silico trained results gradually deviated from the target outputs and eventually misclassified the objects. By contrast, the FFM-designed network accurately made the correct classifications. In addition to the compute-intensive data and error propagation, the loss and gradient calculations can also be carried out through onsite optical and electronic processing (Supplementary Note 13).

We further propose nonlinear FFM learning. As shown in Fig. 2f, in the data propagation, the output is nonlinearly activated before being fed into the next layer, with input to the nonlinear activation recorded and the associated gradient calculated. In the error propagation processes, the input is multiplied with the gradient before propagation. Because only forward propagation is required, the proposed nonlinear training paradigm of the FFM suits general nonlinear functions that are measurable, and is thus applicable to optoelectronic and all-optical nonlinear ONNs^42^. In the experiment, a four-layer ONN was constructed, while the nonlinear activations were electro-optically implemented with an intensity-induced phase nonlinearity $f=x{e}^{{\rm{j}}{|x|}^{2}}$ (ref. ^43^). As shown in Fig. 2g, the classification accuracy of nonlinear ONN increased from 90.4% to 93.0%. Comparatively, we employed a hybrid training method by performing the forward propagation onsite and back-propagation offline^24^, which achieved accuracies of 89.4% and 89.6% with linear and nonlinear ONNs. The four-layer nonlinear FFM learning was further experimentally evaluated with another numerical photorefractive all-optical nonlinear function $f=x{e}^{{\rm{j}}{\rm{\pi }}{|x|}^{2}/(1+{|x|}^{2})}$ (refs. ^30,44,45^) on more challenging CIFAR and ImageNet four-category classification tasks, reaching 60.0% and 59.5% accuracies, respectively. By training the nonlinear ONNs in batch, the error propagation of the nonlinear training can be streamlined with high parallelism, consuming only 1 to around 1.7 times the data propagation time (Supplementary Note 11).

## All-optical imaging and processing with FFM

**Fig. 3:**
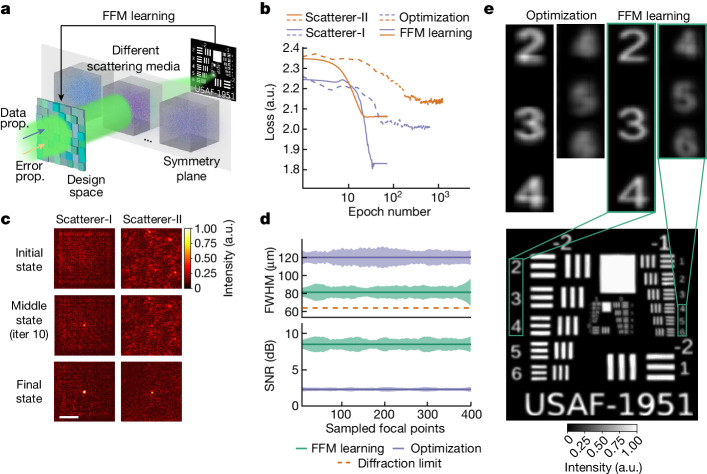
Reaching the diffraction limit through scattering media with FFM learning. **a**, The symmetry of the light propagation with respect to the central plane of the complex medium facilitates FFM learning of focusing through scattering media. **b**, Focus designing with FFM learning and PSO optimization. **c**, FFM-designed focus evolution with different scattering media. **d**, The FWHM and PSNR of the uniformly sampled foci within the 3.2 mm × 3.2 mm design area, designed with the FFM and PSO, respectively. **e**, Imaging resolution calibration on a USAF (the United States Air Force) resolution chart. Scale bar, 1 mm. prop., propagation; iter, iteration. Source Data

Because it takes place on the original physical system, FFM learning overcomes the limitations imposed by offline numerical modelling and simplifies the design of intricate photonic systems. Fig. 3a illustrates the implementation of a point-scanning scattering imaging system. Conventionally in adaptive optics, heuristic optimization methods have been used to optimize the focal points^46^. In this study, we analysed different state-of-the-art optimization methods and utilized particle swarm optimization (PSO) for comparison (Fig. 3b; results from other optimization methods are also presented in Supplementary Fig. 7 and described in Supplementary Note 7). Two distinct types of scattering media, namely the random phase plate (referred to as Scatterer-I) and scotch tape (referred to as Scatterer-II), were employed for evaluation purposes. The gradient-based FFM learning demonstrated higher efficiency, converging after 25 design iterations in both experiments, with converging loss values of 1.84 and 2.07 in two scattering types. By contrast, the PSO method required at least 400 design iterations to converge, yielding final loss values of 2.01 and 2.15. The evolution of FFM self-design is depicted in Fig. 3c, showcasing the gradual convergence of initially randomly distributed intensity profiles into a tight spot. Subsequently, the designed focal points were learned across the entire 3.2 mm × 3.2 mm imaging area. A 20 × 20 array of scanning points was sampled and is shown in Supplementary Fig. 9. The full-width at half-maximum (FWHM) and peak signal-to-noise ratio (PSNR) indices of the optimized focal spots using the FFM and PSO are compared in Fig. 3d. With the FFM, the average FWHM was 81.2 µm, and the mean PSNR was 8.46 dB, with the lowest FWHM achieved being 65.6 µm. Working with a 3.2 mm-wide square aperture and 0.388 m propagation distance, the focal point size designed by FFM learning was approaching the diffraction limit of 64.5 µm (Methods). By contrast, the PSO optimization yielded an FWHM of 120.0 µm and a PSNR of 2.29 dB.

We then evaluated the performance of the designed focal point array in scanning a resolution chart positioned behind the scattering media (Fig. 3e). When the scanning points optimized with PSO were used, digits with a width of 238.7 µm were only vaguely distinguishable (top left panel), while digits with a width of 145.6 µm became completely obscured. By contrast, the scanning foci optimized with the FFM exhibited the capability to resolve line pairs with separations as small as 56.0 µm and linewidths of 64.0 µm (top right panel of Fig. 3e; also see illustrations in Supplementary Fig. 12), which aligns well with the resolution limits of the imaging configurations. The processes of focus optimization and point-scanning are visualized in Supplementary Video 1.

**Fig. 4:**
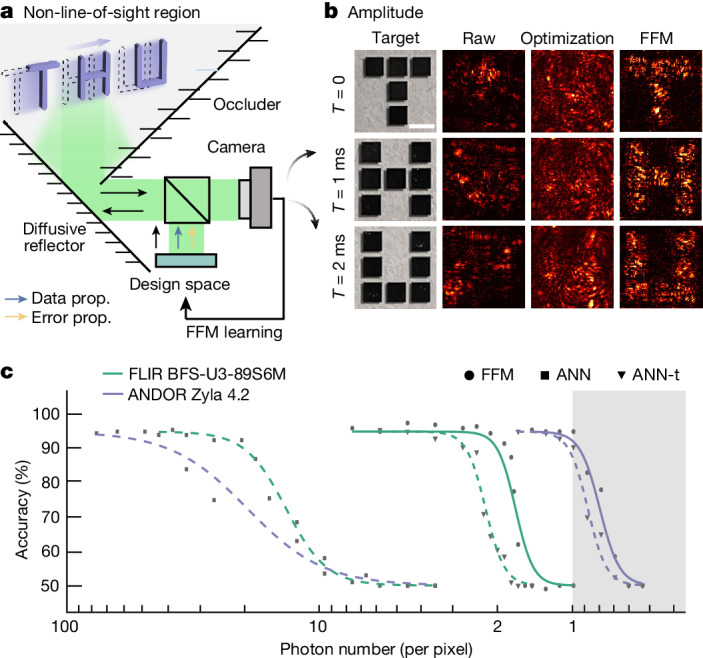
Parallel imaging and all-optical classification of dynamic NLOS scenes with FFM learning. **a**, The FFM learning system setup. **b**, Parallel imaging of dynamic chromium target in the NLOS region. **c**, Photon-efficient all-optical NLOS processing. Scale bar, 1 mm. Source Data

Onsite FFM learning offers a valuable tool for designing nonconventional imaging modalities, particularly in scenarios where precise modelling is impossible, such as non-line-of-sight (NLOS) scenes, where objects are concealed around corners and remain invisible to the observer. Exploiting the spatial symmetry exhibited between the optical paths to and from the hidden objects (Fig. 4a), FFM learning enables all-optical onsite reconstruction and analysis of the dynamic hidden objects. Figure 4b illustrates NLOS imaging. In the learning process, the input wavefront was designed to project all the grids in the object to their target positions simultaneously (Supplementary Fig. 13). Consequently, the hidden objects were recovered in parallel. We used letter-shaped hidden chromium targets ‘T’, ‘H’ and ‘U’. We set the exposure time to 1 ms and the light power to 0.20 mW, enabling the sensor to instantly image the targets as they traversed the field of view. Without the FFM-designed wavefront, the resulting images were severely distorted (‘Raw’ column). Although the PSO optimization improved the signal intensity (third column of Fig. 4b), the objects remained difficult to discern, as indicated by an average SSIM index of 0.36. By contrast, the FFM-designed wavefront recovered the shapes of all three letters, achieving an SSIM of 1.0 for each target. The entire FFM learning process and real-time dynamic target imaging experiment are visualized in Supplementary Video 2.

In addition to dynamic imaging capabilities, the FFM learning approach allows for the all-optical classification of hidden objects in the NLOS region. We compared the classification performance of the FFM with that of an ANN (Supplementary Note 7). To evaluate the photon efficiency of the system, we configured it with varying numbers of incident photons per sensor pixel. We employed a low-noise scientific complementary metal oxide semiconductor (sCMOS) sensor (Andor Zyla 4.2) along with an industrial complementary metal oxide semiconductor (CMOS) sensor (FLIR BFS-U3-89S6M). The results are shown in Fig. 4c, where experimental data points are plotted with curves fitted from the theoretical noise model. We observe that with sufficient photons, the FFM and the ANN had similar performances: FFM learning (ANN) achieved 95.5% (94.5%) and 96.0% (94.0%) accuracies with sCMOS and CMOS sensors. However, the photon number is often limited—for example, with many reflections or highly diffusive surfaces. Under these cases, FFM learning adaptively corrects wavefront distortions and requires fewer photons for accurate classification. Specifically, we set 94.5% accuracy (decreasing by about 1% from the best accuracy) as a turning point and counted the maximal required photon number. The ANN with CMOS and sCMOS required 33.1 and 37.6 photons, respectively. Even when the ANN was trained with the corresponding noise-distorted data (denoted by ANN-t in Fig. 4c), they still required 4.46 and 1.27 photons per pixel to reach the turning point, respectively, while the FFM with CMOS and sCMOS required merely 2.10 and 0.99 photons, equivalent to 5.40 × 10^18^ OPS/W energy efficiency. The outputs are also illustrated in Extended Data Fig. 2. We also observed that the onsite FFM learning is faster to converge, which allows instant error correction (Extended Data Fig. 4).

## Onsite photonic integrated circuits with FFM

**Fig. 5:**
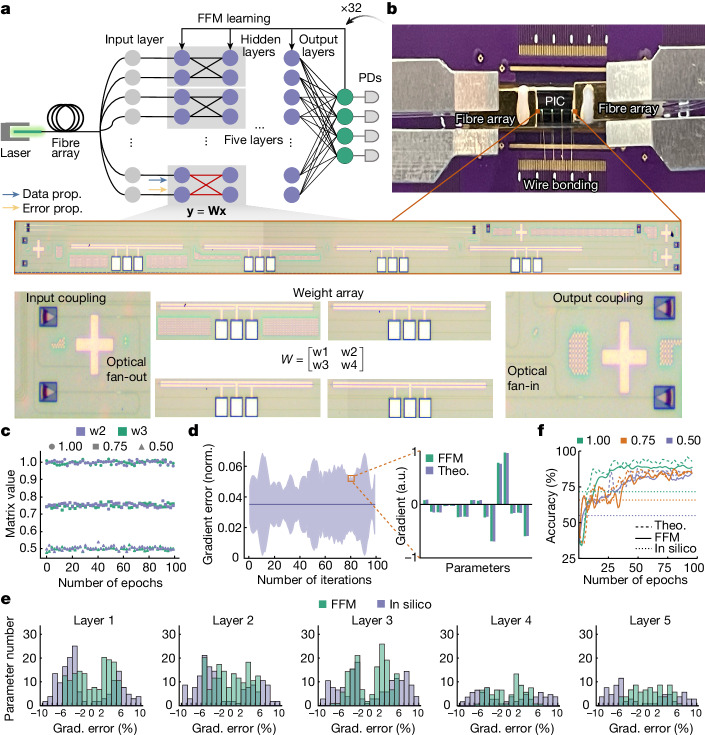
Onsite machine learning with PICs. **a**, Symmetrical integrated photonic neural network architecture. Bottom, microphotographs of the symmetry core and the processing elements. **b**, Experimental system. **c**, Fidelity of the programmed matrix values in the symmetry core. **d**,**e**, Experimental and theoretical network gradients (**d**), with each layer-wise error visualized (**e**). **f**, Accuracy evolution with different configurations of the symmetry core. Scale bar, 1 mm. norm., normalized; PD, photodiode; Theo., theoretical results; Grad., gradient. Source Data

The FFM learning approach can be extended to the self-design of integrated photonic systems. Figure 5a illustrates the implementation of FFM learning using an integrated neural network composed of symmetrical photonic cores connected in series and parallel configurations. The symmetry of the matrix allows for equivalence between the error propagation matrix and the data propagation matrix. Consequently, the data and error propagation share the same direction of propagation. Figure 5b showcases the implementation of the symmetry core and the experimental testing setup for the packaged chip. The symmetry core was realized using a photonic integrated circuit (PIC) fabricated in a silicon-on-insulator silicon photonic foundry. Grating coupler arrays were utilized to couple the input light into the chip. As depicted at the bottom of Fig. 5b, each input is distributed using on-chip beam splitters and combined using 2 × 1 multimode interferometers. The matrix elements were implemented by configuring the attenuation coefficient of on-chip variable optical attenuators (VOAs) through forward biasing the p–i–n junction with varying levels of injection currents. The electrodes of the VOA arrays were routed and wire-bonded to a printed circuit board for matrix reconfiguration, and the light is coupled to and from the PIC using packaged fibre arrays. Further details of the testing system and packaging setups can be found in the Methods and Supplementary Note 6.

The constructed neural network was utilized for the classification of standard Iris flower data, with inputs processed into a 16 × 1 vector and the outputs representing one of three flower categories. During training, the symmetry matrix was configured to one of three symmetry ratios by setting the weights $w3/w1$ to 1.0, 0.75 or 0.5. The fidelity of the matrix programming during training is visualized in Fig. 5c, with the standard deviation of the temporal drifting of the three symmetry matrix values recorded as 0.012%, 0.012% and 0.010%, respectively. Under this level of uncertainty, the experimental gradient was compared to simulation values. As depicted in Fig. 5d, the experimental gradients display an average deviation of 3.5% from the ideal simulation values. The inset of Fig. 5d illustrates the designing gradients of the second layer at the 80th learning iteration, while the error of the entire neural network is visualized in Fig. 5e. At iteration 80, FFM learning (in silico training) exhibited gradient errors of 3.50% (5.10%), 3.58% (5.19%), 3.51% (5.24%), 3.56% (5.29%) and 3.46% (5.94%). The evolution of the design accuracies is depicted in Fig. 5f. Both the ideal simulation and FFM experiments required approximately 100 epochs to converge. Under three symmetry ratio configurations, the experimental performance was similar to that of the simulation, where the network converged to accuracies of 94.7%, 89.2% and 89.0%. The FFM approach achieved accuracies of 94.2%, 89.2% and 88.7%. For comparison, the in silico designed network exhibited experimental accuracies of 71.7%, 65.8% and 55.0%. We have further evaluated networks with symmetrical propagation and networks with full connection, and they show similar classification performances when numbers of learnable weights are close (Supplementary Note 12).

The ability to delicately design gain/loss in the optical system facilitates manipulation of a non-Hermitian open system at will. Here we numerically demonstrated that the FFM can self-design non-Hermitian traversing over the exceptional point without resorting to physical models. The details are revealed in the Methods.

## Discussion

Taking advantage of high-speed, model-free photonic machine learning, FFM learning can simultaneously improve the design speed and performance of an optical system beyond what can be achieved by electronic circuit-based computers. As summarized in the Methods and Supplementary Note 9, FFM learning not only experimentally constructs the deepest photonic neural networks with an accuracy equivalent to that of the ideal model, but also enhances the resolution of scattering imaging, approaching the diffraction limit. Moreover, it enables millisecond parallel imaging and achieves exa operations per second per watt (exaOPS/W) high-energy efficiency processing of hidden objects beyond the line of sight. Taking advantage of commercially available high-speed optical modulators and detectors, the implementation of free-space and integrated FFM learning is poised to surpass GPU (graphics processing unit)-accelerated learning implementations. Considering the ongoing advancements in optoelectronic device development, substantial improvements in high-speed FFM learning are anticipated in the near future, potentially reaching efficiencies orders of magnitude higher.

To summarize, we have proposed and experimentally demonstrated the FFM gradient descent methods for self-designing the free-space and integrated optical systems. Because of the ubiquitous noise and universal uncertainty in the physical nature, offline designed physical AI methods are inevitably imperfect. The proposed onsite machine learning approach, however, alleviates the constraints of numerical modelling and realizes high-performance general optical AI and AI for optics. Our work shows that both free-space and integrated optical systems can be parameterized as an onsite neural network and efficiently self-designed. These findings imply that optical systems fundamentally embody differentiable learnable neural architectures, providing a new route to model-free high-performance self-design of optical systems and self-learning physics, and suggesting the possibilities of large-scale, high-efficiency physical AI in the post-Moore era^47^.

## Methods

### Experiments

#### Free-space experimental system

In the free-space FFM system, the light source of the optical computing was generated using a solid-state laser (MFL-FN-532) at a working wavelength of 532 nm. The coherent light wavefront was expanded using a beam expander and then split into two paths by a beam splitter (BS013, Thorlabs). One path was directed towards spatial light modulators (SLMs) for data/error complex field loading, while the other path served as the interference beam with light intensity attenuated by two polarizers (LPNIR100-MP2, Thorlabs). The SLM for field loading (X15213-01, Hamamatsu Photonics) was configured to work in the amplitude modulation mode, which consists of 1280 × 1024 modulation elements with a pitch of 12.5 µm and 8-bit programming depth. It can modulate incident light at a maximum speed of 60 Hz. The amplitude modulated optical field was then 1:1-relayed to the design space SLM (E-Series, Meadowlark Optics) by 4 *f* system for phase modulation, after polarization adjustment with a half-wave plate and a linear polarizer. The design space SLM was a reflective liquid crystal on silicon device with a high zero-order diffraction efficiency of 91%. It contained 1920 × 1200 modulation elements, each with a size of 8 µm and 8-bit precision, and operated at a maximum frame rate of 60 Hz, with an update time of 17 ms. The phase modulation SLM worked both for electric field loading and design phase loading. The wavefront carrying the input light field and the design phase then probed the symmetry propagation system to perform specific tasks. The output beam was split towards two separate sensors before propagating over a length of 0.388 m. A CMOS sensor (BFS-U3-89S6M, FLIR) was utilized to measure the complex field, with decent quantum efficiency (63.99%) and low dark noise (2.47 e^−^). Two sensors were employed to respectively measure the amplitude and phase of the results. The CMOS sensor can achieve a maximum frame rate of 42 Hz with pixel settings of 4096 × 2160, where each pixel has a size of 3.45 µm and the readout values are configured as 8-bit. To measure the complex field, we employed phase-shifting holography, where the phase difference between the reference beam and signal beam is shifted 0,0.5π radian, and 1.0π radian, for the reconstruction of the phase and amplitude of the signal light. A free-space optical system is illustrated in Supplementary Fig. 15. In photon number-limited all-optical NLOS processing, a scientific CMOS sensor Andor Zyla 4.2 with 0.9 e^−^ read noise and 82% quantum efficiency was used for evaluation. The photon number was controlled with a variable neutral density filter.

We configured 2/4/6/8 layers of networks, with a propagation distance of 0.388 m for each layer. For imaging through scattering experiment, the distance between the modulator and the scattering medium was 0.194 m, while the distance between the scattering medium and the output sensor was also 0.194 m. The computational operations within each layer of the ONN in section consisted of two stages (Fig. 2a): optical modulation in the design region and fixed free-space propagation. Taking advantage of the symmetry of free-space propagation (indicated by dashed grey lines), the FFM learning method is employed to optimize the effective index of the liquid crystal in the spatial modulators. The liquid crystal is configured in the pure-phase module; hence, the optimization is in essence on the real parts of the effective refractive index *n*_*R*_. Specifically, in the design process, the data field (shown in blue) undergoes propagation through the physical systems. The resulting outputs from this data propagation are compared with the target outputs to generate error fields (represented in orange). These error fields are then forward propagated through the symmetrical system to obtain error field outputs. Gradients of each designed layer are computed using the data inputs and error outputs. Before FFM learning is conducted, spatially symmetrical reciprocity is validated through a two-step propagation (Supplementary Note 4). The experimental setup illustrated in the bottom panel of Fig. 2a implements layer-by-layer data and error propagation with a 0.388 m propagation distance and 160,000 optical neurons (400 × 400). The complex output light field is probed by interfacing the signal beam with a reference beam, and phase-shifting holography is employed to retrieve the complex light field. The ground truth target was set as the one-hot encoding with the ones designating the right categories. In the error propagation, layer parameters are loaded in reverse order.

For results in Fig. 3, the input wavefront covering an area of 3.2 mm × 3.2 mm was divided into 400 × 400 pixels and concurrently optimized to achieve focused imaging through the scattering media. The designed focal points were subsequently utilized to evaluate point-scanning of an object located beyond the scattering media. The resolution was calculated from $d=0.5\,\lambda /{\rm{N}}{\rm{A}}$ (ref. ^48^), with NA standing for numerical aperture; see also the illustrations in Supplementary Fig. 11. Two types of scattering media were used in the experiment. The first type was generated using a kilohertz high-speed phase modulator projected with a random phase pattern. The degree of scattering can be adjusted by binning the granularity of the scattering patterns into different sizes. The second type of scattering medium was made of a used scotch tape (Supplementary Fig. 8). In the dynamic parallel NLOS experiment, the distance between the modulator and the objects in the scene, and the distances between the objects and the camera were both 0.194 m. Two different NLOS systems were used in the experiment. For results in Fig. 4b, the input and output light were double reflected by a diffusive reflector, with the ‘T’, ‘H’ and ‘U’ targets placed 0.04 m away (Supplementary Fig. 16). For phase imaging and all-optical processing, to accommodate the large training data number, the diffusive reflector and the targets were projected with another SLM. All the experiments had 8 µm neurons with neuron number of 400 × 400.

Additionally, in Extended Data Fig. 4a, we illustrate the application of FFM learning for imaging phase images with a resolution of 28 × 28. Through FFM learning, the phase targets projected with SLM at the NLOS region are converted into intensity images on the camera at the line-of-sight (LOS) region. Alongside FFM learning, an ANN for image reconstruction (Supplementary Note 7) was trained for the same task, with four handwritten digits employed for learning. At the bottom of Extended Data Fig. 4a, we characterize the structural similarity between the recovered image and the original image under different signal-to-noise ratio levels. With a 15 dB signal-to-noise ratio, FFM learning achieved an SSIM index of 0.81, while the ANN only recovered an image with a 0.40 SSIM similarity. Furthermore, we employed the rotation of the scattering phase around its central point (Supplementary Fig. 14). The system operates at two rotating speeds, denoted as *v*_1_ = 1.0 degrees per iteration and *v*_2_ = 2.5 degrees per iteration. The results are shown in Extended Data Fig. 4b; with the low rotating speed, the in silico ANN eventually converged to the same accuracy as the FFM, albeit requiring a larger number of iterations. As the rotational speed increased from *v*_1_ to *v*_2_, (ν, rotation velocity) the ANN model synchronously degraded with changes in the scattering medium. By contrast, FFM learning continued to advance its accuracy as the learning process evolved.

#### Integrated photonic experiment system

The integrated photonic chip was tapped out in a commercial silicon photonics foundry. To facilitate an end-to-end incoherent matrix-vector multiplication, the chip realizes a photonic core, incorporating four grating coupler arrays for on- and off-chip optical coupling, 220 nm fully etched strip waveguides for signal routing, four multimode interferometers for optical fan-in and fan-out, and four p–i–n VOAs for weight programming.

The die cores were first diced from the fabricated 8 in. wafer. Then optical inputs and outputs (I/Os) were packaged with fibre arrays and electrical input pads were bonded off-chip to a dedicated printed circuit board. The chip under the packaging was shown in Fig. 5b. Details of the packaging methods are also described in Supplementary Note 6. After packaging, in the integrated photonic experiment system, as illustrated in Extended Data Fig. 3, two lasers with output powers of 10 dBm were utilized as two-channel input sources. For incoherent matrix operations, one laser operated at a precise wavelength of 1,550 nm, while the other operated at 1,551 nm. The polarization of light within the fibre was adjusted using polarization controllers, ensuring its alignment with the transverse electric coupling mode of the grating coupler. Following the polarization control, two microelectron-mechanical-systems variable optical attenuators (V1550A, Thorlabs) were employed to encode the input signal and the weight of the imaginary refractive index. The outputs of the variable optical attenuators were then coupled into the photonic chip through fibre arrays. On the chip, four p–i–n VOAs controlled by an arbitrary waveform generator attenuated the fanned-out two-channel input light, and fanned-in the signal into two separate output paths. The attenuated light was subsequently coupled off-chip and detected by 5 GHz photodiodes with around 1.0 A/W calibrated photoresponsivity over the 1,500–1,600 nm optical band. The captured photocurrents were amplified with transimpedance amplifiers (AMP100, Thorlabs) of 100 kΩ transimpedance, and the amplified output voltage signals were finally measured and read out by a multichannel oscilloscope.

This network presented in the main text comprises five layers with neuron counts of 16, 16, 16, 8 and 8, respectively, from the first to the last layer. Within each layer, input data are partitioned into pairs, each containing two elements. These input pairs are encoded using mutually incoherent wavelengths and passed through the symmetrical cores. The resulting outputs are detected using high-speed photodiodes, where the mutually incoherent signals are added together. Consequently, each core performs a matrix-vector multiplication operation. To facilitate interlayer connections, the outputs of the neurons are shuffled before being connected to the subsequent layer.

#### The FFM learning procedure

In both free-space and integrated systems, FFM learning unfolds sequentially into four steps: data propagation, final loss calculation, error propagation and gradient calculation. As elaborated in Supplementary Note 5, the complete FFM learning protocol entails a synergy between optical and electronic systems, with compute-intensive propagations executed on site. On the electronic front, it oversees optoelectronic nonlinear activation between layers, final loss and gradient calculation, and input data loading. Furthermore, we have confirmed that loss and gradients can be computed using onsite optics and electronics, as outlined in Supplementary Note 13. Additionally, the nonlinear function can be realized entirely optically or optoelectronically without the need for digital electronics.

### Dataset preparation

#### MNIST dataset

The MNIST dataset^49^ is a collection of ten-category handwritten digits, comprising 60,000 training samples and 10,000 testing samples. Each original sample was converted into an image with a resolution of 392 × 392 and then zero-padded to a resolution of 400 × 400. In the multilayer classification experiment, each sample in the ten categories was utilized to construct the input complex field with a phase set to zero. In the NLOS tasks, the samples were treated as invisible objects and modelled as a phase-only mask for the imaging and classification.

#### Fashion-MNIST dataset

The Fashion-MNIST dataset^50^ comprises fashion products of ten different categories, consisting of a training set with 60,000 samples and a test set with 10,000 samples. Each original sample was transformed into an image with a resolution of 392 × 392 and then padded to a resolution of 400 × 400. In the multilayer classification experiment, each sample was used to create the input complex field with a phase set to zero.

#### CIFAR-10 dataset

The CIFAR-10 dataset^51^ is a subset of the 80 million tiny images dataset, containing 50,000 training images and 10,000 test images. Each original sample was converted into a greyscale image and resized to a resolution of 400 × 400. In the multilayer classification experiment, each sample was used to construct the input complex field with a phase set to zero.

#### ImageNet dataset

The ImageNet dataset^52^ is an image database organized according to the WordNet hierarchy, in which each node of the hierarchy is depicted by hundreds and thousands of images and in total there are 120 million images for training and 50,000 images for testing. Each original sample was converted into a greyscale image and resized to a resolution of 400 × 400. In the multilayer classification experiment, each sample was used to construct the input complex field with phase values set to zero.

#### MWD dataset

The MWD dataset^53^ comprises weather condition images of four different outdoor scenes: sunrise, shine, rain and cloudy. It contains a total of 1,125 samples, with 800 samples used for training and 325 samples for testing. Each original sample is converted into a greyscale image with a resolution of 400 × 400. In the multilayer classification experiment, these greyscale images were utilized to construct the input complex field, with the phase set to zero for all samples.

#### Iris dataset

The Iris dataset^54^ consists of three Iris species, with 50 samples for each species, along with several properties describing each flower. One of the flower species is linearly separable from the other two, while the other two species are not linearly separable from each other. For our experiment, we used 120 samples, with 40 samples from each species, for training, and 30 samples for testing. Each original sample in the dataset contains four entries that describe the shape of the Iris species. In the PIC experiment, each entry was duplicated to create four identical data points, resulting in a total of 16-channel input data.

#### Chromium target dataset

The real scene dataset is specifically designed for NLOS scenarios involving real-world objects. It consists of glass chrome plates with distinct regions: reflective and translucent. The reflective areas represent the physical scene itself (letter targets ‘T’, ‘H’ and ‘U’). During the training process, a single reflective area is used, and it is systematically moved within the same plane to generate nine different training scenes. The test data consist of three letters composed of multiple reflective areas. During testing, the scene plate was translated with motor stage to position different letters within the illuminated regions.

### Embedded photonic neural network architecture

#### Free-space neural network

The input data $X\in {C}^{\mathrm{160,000}}$; the input is per-point weighted by designed complex weight $M\in {C}^{\mathrm{160,000}}$ and propagated to the outputs. Denote the propagation matrix as $T\in {C}^{\mathrm{160,000}\times \mathrm{160,000}}$; each layer propagation is $Y=T(M\cdot X)$. In the deep free-space neural network, the network model is $Y=({\Pi }_{i=1}^{L}{T}_{i}{M}_{i})X$, where *L* is the total number of layers. In imaging through scattering media, *Y* *=* *TSTMX*, where *S* represents the transportation of the scattering media. In the NLOS imaging system, $Y={TR}_{{\rm{ref}}}T{R}_{{\rm{ob}}}T{R}_{{\rm{ref}}}TMX$, where *R*_ob_ represents the NLOS object reflectance and *R*_ref_ represents the reflector (refer to Supplementary Note 3 for derivation of symmetry in transportation matrices).

The loss function used in the free-space neural networks is the mean squared error (MSE) loss, which calculates the average squared difference between the predicted outputs and the ground truth. In classification tasks in deep ONNs, for Fig. 2c,d, we respectively used 60,000, 60,000, 30,000 and 800 samples for training, and 10,000, 10,000, 5,000 and 325 samples for testing when using the MNIST, CIFAR-10, Fashion-MNIST and MWD datasets. For Fig. 2f,g, we used 10,000, 8,000 and 5,200 samples for training, and 500, 200 and 200 samples for testing, on MNIST (categories ‘0’, ‘2’, ‘4’, ‘6’, ‘8’), CIFAR (categories ‘automobile’, ‘dog’, ‘frog’ and ‘horse’) and ImageNet (categories ‘tabby’, ‘basketball’, ‘castle’ and ‘envelope’). The targets were configured as binary images where the maximal values are normalized to 1.0 in the detected regions corresponding to the respective categories and 0 in the remaining regions. The MSE loss was employed to measure the discrepancy between the intensity of the output diffraction field and the desired target. When calculating testing accuracy, we tested five times for each experiment and got the mean value of the convergence results. In high-resolution imaging systems, the focusing target was a binary image with a value of 1.0 in the central region of the image, with a diameter of 10 pixels and 0 in the surrounding regions. The intensity of the output field was compared with the desired target to compute the MSE loss. In parallel dynamic NLOS imaging and processing systems, the target for the 3 × 3 square grid array imaging was a binary image with a value of 1.0 (high reflectance) in specific regions of the grid that formed letters such as ‘T’, ‘H’ or ‘U’. In high-resolution phase imaging, the target is obtained by binarizing greyscale images of the object to be detected. For phase object imaging, we selected objects from four different categories—‘0’, ‘2’, ‘7’ and ‘9’—of the MNIST dataset and mapped the pixel values to phase range 0–π. The output targets were the original intensity images. For the phase object classification task in NLOS system, we used two categories, ‘0’ and ‘7’, from the MNIST dataset, 2,000 samples for training and 200 samples for testing. The targets were one-hot vectors similar to those used in deep optical neural networks. The MSE loss was employed to quantify the discrepancy between the predicted intensity outputs and the desired targets for the imaging and classification experiments. The optimizer used to optimize the parameters of free-space neural networks is the Adam optimizer^55^, which incorporates exponentially weighted averaging and accelerates gradient descent.

#### PIC network

In the PIC network, the 16-channel input ${x}_{0}=[{x}_{1},{x}_{2},\ldots ,{x}_{16}]$ is multiplied by a 16-channel modulation mask *M*_1_ and the results are connected to the next layer through a local 2 × 2 matrix. In the 16-channel output ${y}_{1}=[\,{y}_{1},{y}_{2},\ldots ,{y}_{16}]$, each adjacent pair of outputs $[\,{y}_{2n-1},{y}_{2n}]\,(n=\mathrm{1,2},\ldots ,8)$ is only related to the corresponding pair of inputs $[{x}_{2n-1},{x}_{2n}]$, which can be calculated by $$\left[\begin{array}{c}{y}_{2n-1}\\ {y}_{2n}\end{array}\right]=\left[\begin{array}{cc}{w}_{n1} & {w}_{n2}\\ {w}_{n3} & {w}_{n4}\end{array}\right]\left[\begin{array}{c}{x}_{2n-1}\\ {x}_{2n}\end{array}\right]$$. The ${w}_{n2},{w}_{n3}$ of the modulation mask are the same to satisfy the symmetry condition. Afterwards, the 16-channel output undergoes a specific shuffling process. The output is rearranged according to a predetermined table: *T*_1 _= [0,2,1,3,4,6,5,7,8,10,9,11,12,14,13,15] and the resulting arrangement determines the 16-channel input for the next layer by ${x}_{1}[i]={y}_{1}[{T}_{1}[i]]\,(i=\mathrm{1,2},\ldots ,16)$. Layer 2 follows a similar structure as the first layer, and the shuffling table for the output is defined as *T*_2 _= [0,4,8,12, 1,5,9,13, 2,6,10,14, 3,7,11,15]. Accordingly, layer 3 transforms the modulated 16-channel input into an 8-channel output by summing adjacent channel inputs $\left[{x}_{2n-1},{x}_{2n}\right]$ to obtain a single output channel, *y*_*n*_. Then the output is shuffled based on another predefined table *T*_3 _= [0,2,1,3, 4,6,5,7]. Layer 4 is similar to the first layer but has only eight channels, and a new shuffling table is used: *T*_4 _= [0,4, 1,5, 2,6, 3,7]. The last layer, similar to layer 3, converts the modulated eight-channel input into a four-channel output. The final output is used to determine the category corresponding to the input data. The network architecture is visualized in Supplementary Fig. 16.

The loss function of a photonic integrated neural network is cross-entropy loss, which aims to minimize the difference between the predicted probability distribution and the true probability distribution. It can be calculated in the following way:

$$L=\frac{1}{N}\mathop{\sum }\limits_{i=1}^{N}-\log \left(\frac{{e}^{{y}_{5,i}[gt[i]]}}{{\sum }_{k}^{K}{e}^{{y}_{5,i}[k]}}\right)[\,gt[i]]$$

with given final output probability distribution *y*_5_, the target label *gt*, the number of training samples *N*, and the number of categories *K*. In a photonic integrated neural network, the output is the magnitude of the voltage signal received from the oscilloscope and the target is the label of the input data. The dataset contains 120 training samples and 30 testing samples in total. Cross-entropy loss is used to measure the difference between the output and the target, and the loss is optimized with an Adam optimizer.

### Self-designing non-Hermitian system

**Extended Data Fig. 1:**
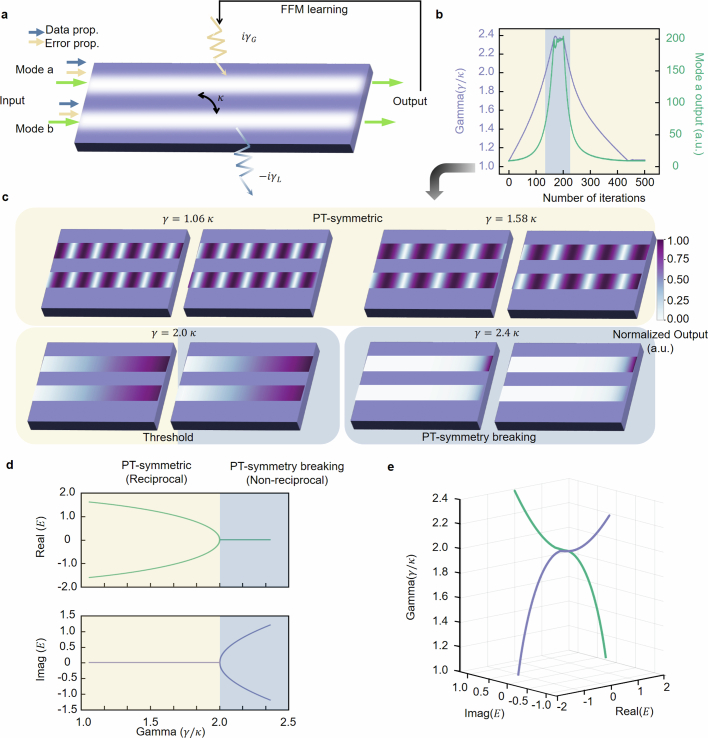
FFM learning to search exceptional points in non-Hermitian system. **a**, The coupled waveguide system with gain ${\gamma }_{G}$ and loss ${\gamma }_{L}$. **b**, The output intensity evolution and the corresponding gain and loss coefficients *γ* during the learning process. **c**, The field distribution at different learning time steps. **d**, The theoretical results of eigen spectrum. **e**, Band braiding in the non-Hermitian system with FFM learning. Source Data

**Extended Data Fig. 2:**
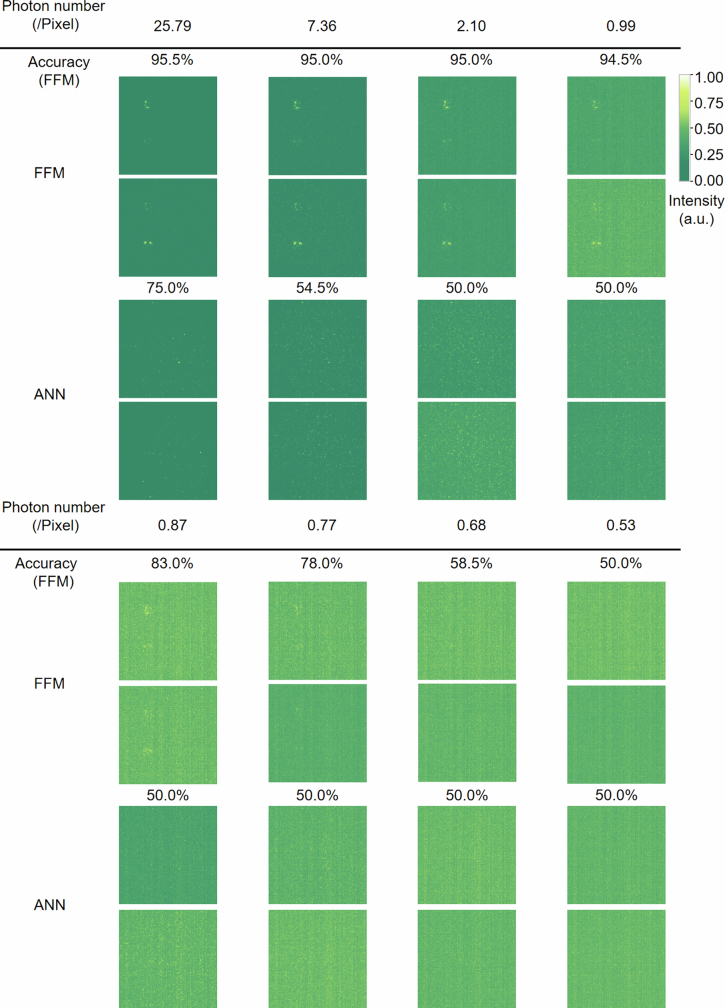
Visualizations of input and output results of FFM learning in NLOS tasks under different signal intensities with sCMOS sensor. Even below the 1 photon/pixel regime, the FFM still achieved classification accuracies of 94.5%. On the contrary, the ANN methods completely failed to classify and produced around 50% accuracies with less than around 7 photons/pixel.

**Extended Data Fig. 3:**
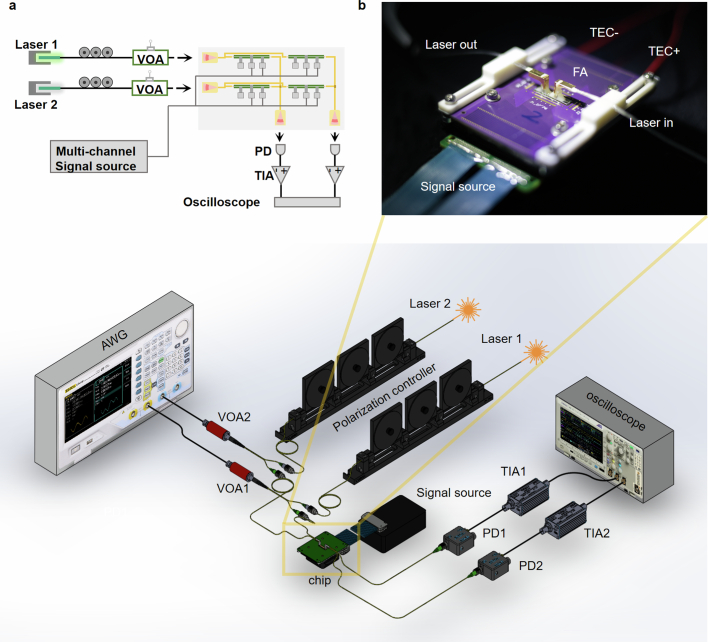
Schematic (a) and system configuration (b) of the PIC experiment. Inset: zoomed-in view of the packaged chip. PC, polarization controller; AWG, arbitrary waveform generator; VOA, variable optical attenuator; PD, photodiode; TIA, transimpedance amplifier.

Traditionally, the study of exceptional points in a non-Hermitian system requires numerically solving an a priori model^56^. Here we numerically demonstrate that the FFM can self-design non-Hermitian traversing over the exceptional point without resorting to physical models. As shown in Extended Data Fig. 1, we study the wave propagation in symmetrical dual waveguides, where one waveguide is for the gain and the other for the loss. In the case studied, while we can probe the system by feeding in optical signals, we do not explicitly know the formulation of the Hamiltonian. To design the gain and loss, we use as a target the breaking of the lateral reciprocity of the system, that is, control the asymmetry of the output signal by feeding input to symmetrical channels but maximize the output of one waveguide (Supplementary Note 8). It was observed that with FFM learning, the system output could switch from a reciprocal to a nonreciprocal regime, or vice versa. When comparing with the knowledge of non-Hermitian optics, we discovered that the switching guided by FFM self-design was accompanied with the spontaneous parity-time symmetry-breaking, and the learning process corresponded to the non-Hermitian braiding group ${{\mathbb{B}}}_{2}$. The FFM design provides a target-oriented way to solve complex topological systems.

### Comparison with state-of-the-art high-performance optical systems

#### Deep ONNs

Realizing neural networks with deeper layers and a larger scale is of vital importance for optical computing to gain higher performances. As shown in Extended Data Table 1, the depth of reconfigurable optical neural networks reported in previous works is at most five for free-space neural networks and three for PICs^17–19,24,34,38^. Further increasing the network depth would cause error accumulation and degrades the performances of the neural networks^24^. The FFM onsite machine learning method alleviates the necessity of a numerical model, and thus is without the issues of imprecise modelling. Accordingly, FFM learning achieved ONNs with a depth of at least eight for free-space neural networks with millions of parameters, and reached a depth of five for integrated photonic circuits. During FFM learning, the network performances stably increase. A more detailed discussion on emerging neural network training methods^57,58^ is presented in Supplementary Note 10.

#### Focusing through scattering media

Focusing through scattering media with high precision has applications in microscopic as well as macroscopic imaging^59,60^. Existing focusing through scattering methods relies on optimization with feedback, which is not optimal and is slow to converge^61^. FFM learning transforms the focusing system into a target-oriented onsite neural network and learns to focus with gradient descent. Our results reach the theoretical limit of optical resolution.

#### NLOS scene imaging and processing

NLOS is a difficult scenario whereby objects are hidden beyond the line of sight. As shown in Extended Data Table 2, due to the lack of direct photons, imaging is challenging in NLOS and requires dedicated scanning such that the speed is usually limited to seconds to minutes per frame^62–64^. FFM learning inherently models the highly diffusive propagation into part of the imaging system, and enables parallel imaging of the object at a single shot. Our system demonstrates 1 ms exposure time, but the speed can be further accelerated with high-speed cameras. FFM learning can also transform the NLOS propagation into part of the onsite processing system, so that the hidden object can be all-optically processed with as few as <1 photon per pixel.

#### Exceptional point searching

FFM learning supports analysis of the topological photonics system on site. We achieved results on a non-Hermitian system that agreed well with the theoretical analysis. Previous studies needed to first model the system offline and carefully approach the theoretical results on site by carefully tuning the gain and loss^56,65–67^. Our findings show that the symmetry-breaking of the non-Hermitian system can also be realized on site without careful offline modelling, which presents a new way to study optics and may help in designing topological systems of larger scale and higher order.

## Online content

Any methods, additional references, Nature Portfolio reporting summaries, source data, extended data, supplementary information, acknowledgements, peer review information, details of author contributions and competing interests, and statements of data and code availability are available at 10.1038/s41586-024-07687-4.

### Supplementary information

Supplementary Information

Supplementary Video 1 A video displaying the process of imaging through scattering media by scanning the image plane with local focal point and comparing the imaging results of FFM learning with optimization method.

Supplementary Video 2 A video displaying the process of imaging in NLOS scenes. It shows the learning process with square grid regions and testing process of imaging dynamic chromium letter targets (‘T’, ‘H’ and ‘U’).

### Source data

**Extended Data Fig. 4:**
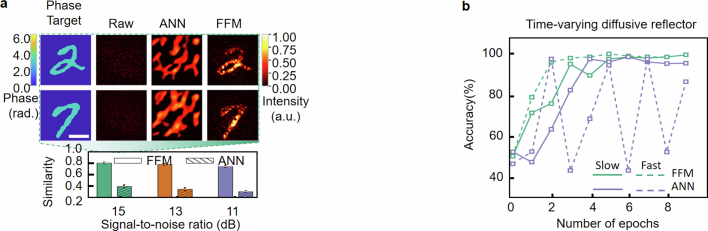
Parallel imaging and all-optical classification of dynamic NLOS scenes with FFM learning. **a**, Parallel imaging of phase target in the NLOS region. Similarity: SSIM. **b**, Fast FFM learning with time-varying diffusive reflector. Scalebar: 1 mm. [Optica, 2022]^38^, [Nature, 2021]^24^, [Nat. Photonics, 2021]^18^, [Science 2023]^34^, [Nature 2022]^19^, [Nat. Photonics, 2017]^17^. Source Data

Source Data Figs. 2–5 and Source Data Extended Data Figs. 1 and 4

## Figures and Tables

**Extended Data Table 1:**
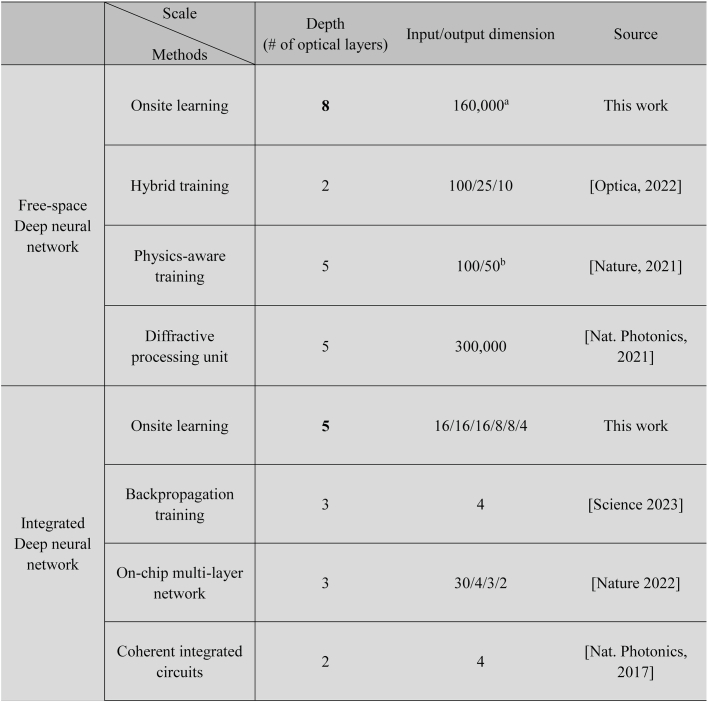
Deep ONNs

a) Each optical layer performs 160,000-D to 160,000-D linear connection and optoelectronic nonlinear activations.

b) Each optical layer nonlinearly transformed 100-D input to 50-D output, with linear transformations executed on electronic computer.

[Science, 2021]^62^, [Nat. Photonics, 2022]^63^, [Nature, 2018]^64^.

**Extended Data Table 2:**
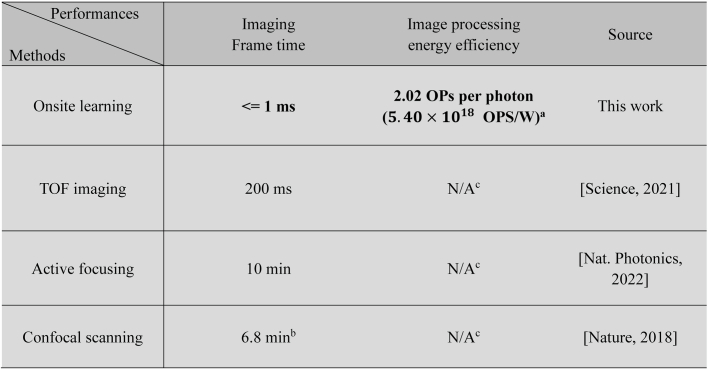
NLOS scene imaging and processing capabilities

a) With an input dimension of *N*_*in* _*=* 400 × 400 and output dimension *N*_*out* _*=* 2, the onsite machine learning executed a fully connected layer with $\sim ({N}_{{in}}{N}_{{out}})$ operations (OP). Considering the consuming energy $E={N}_{{in}}{kh}\nu$ (*k* *=* 0.99), the energy efficiency gives $\eta ={N}_{{in}}{N}_{{out}}/({N}_{{in}}{kh}\nu )$.

b) Reconstruction overhead not included.

c) Incapable of all-optical processing.

## Data Availability

The data are available as part of the Supplementary files. Source data are provided with this paper.

## Code availability

The software code is available at Zenodo (10.5281/zenodo.10820583)^68^.
