# Supplementary material for: Fully forward mode training for optical neural networks
Source: Nature. 2024 Aug 7;632(8024):280–6. doi: 10.1038/s41586-024-07687-4 (PMC11306102; doi:10.1038/s41586-024-07687-4)
Supplement: Supplementary file 1 — Supplementary Information [file 41586_2024_7687_MOESM1_ESM.docx]

**Supplementary Information**

[Supplementary Note 1. Mapping optical system to onsite differentiable neural network 3](#_Toc154752996)

[Supplementary Note 2. Derivation of fully forward-mode gradient descent 4](#_Toc154752997)

[Supplementary Note 3. Spatial symmetries in free-space and integrated photonics 5](#_Toc154752998)

[Supplementary Note 4. Validation of spatial symmetric reciprocity via two-step propagation 7](#_Toc154752999)

[Supplementary Note 5. FFM learning protocol 9](#_Toc154753000)

[Supplementary Note 6. PIC chip packaging 14](#_Toc154753001)

[Supplementary Note 7. Methods for comparison 14](#_Toc154753002)

[Supplementary Note 8. Model-free non-Hermitian topological band braiding in the photonic integrated circuits 16](#_Toc154753003)

[Supplementary Note 9. Summary of the FFM learning 17](#_Toc154753004)

[Supplementary Note 10. FFM and other emerging neural network training methods 18](#_Toc154753005)

[Supplementary Note 11. Parallel multilayer implementation of FFM learning and incorporation of nonlinear activation 20](#_Toc154753006)

[Supplementary Note 12. Symmetrical neural networks 21](#_Toc154753007)

[Supplementary Note 13. Onsite calculation of loss and gradient 22](#_Toc154753008)

[Supplementary Tables. 23](#_Toc154753009)

[Supplementary Figures S1-S18 25](#_Toc154753010)

[Supplementary video descriptions 47](#_Toc154753011)

[References 48](#_Toc154753012)

# Supplementary Note 1. Mapping optical system to onsite differentiable neural network

The basic ingredients of an optical system are the (real) refractive index, the gain and loss, which can be summarized as the real and imaginary parts of the refractive index: $n=n_{R}+in_{I}$. In this section, we will prove two conclusions. First, the linear propagation of the optical system can be reformulated as a linearly connected neural network. Secondly, the neural network is differentiable so as to design the system parameters with gradient descent.

Consider the optical system governed by Maxwell equation, $(\nabla\times\nabla\times\boldsymbol{-}\mu_{0}\epsilon_{0}\omega_{0}^{2}\epsilon_{r}\boldsymbol{)E=}-j\mu_{0}\mu_{r}\omega_{0}\boldsymbol{J}$**,** with $\boldsymbol{E}$ the electric field distribution, $\boldsymbol{J}$ the electric current source, and $\epsilon_{0},\epsilon_{r},\mu_{0},\mu_{r}$ the vacuum and relative permittivity and permeability respectively. The Maxwell equation can be vectorized as $A\left( \epsilon_{r} \right)\boldsymbol{E=}\mu_{r}\boldsymbol{J}$. As the considered scenario is seeded by the same current source $\boldsymbol{J}$, the input electric field $\boldsymbol{E}_{\boldsymbol{i}}$ and the output electric $\boldsymbol{E}_{\boldsymbol{o}}$ both satisfy $A_{i}\boldsymbol{E}_{\boldsymbol{i}}\boldsymbol{=}\mu_{r}\boldsymbol{J}$ and $A_{o}\boldsymbol{E}_{\boldsymbol{o}}\boldsymbol{=}\mu_{r}\boldsymbol{J}$. We then set out to prove that in a linear system,

*Proposition*: there exists a pseudo inverse matrix $A_{o}^{+}$, such that $A_{o}^{+}A_{o}=\boldsymbol{I}$**,** where $\boldsymbol{I}$ is the identity matrix.

*Proof*: Let $\boldsymbol{J\in}C^{N_{j}}$**,** $\boldsymbol{E}_{\boldsymbol{o}}\boldsymbol{\in}C^{N_{o}}$ **,** and $\boldsymbol{j}_{\boldsymbol{i}}$ the one-hot vector with non-zero entry at the ***i****-th* position, then $\boldsymbol{j}_{\boldsymbol{i}}$ would be the base current source that ${\boldsymbol{J=}\boldsymbol{\Sigma}_{\boldsymbol{i}}\alpha_{i}\boldsymbol{j}}_{\boldsymbol{i}}\boldsymbol{.}$ For every $\boldsymbol{j}_{\boldsymbol{i}}$ as the source of the system, denote the output electric field distribution as $\boldsymbol{e}_{\boldsymbol{i}}\boldsymbol{(}\boldsymbol{e}_{\boldsymbol{i}}\boldsymbol{\in}\boldsymbol{C}^{\boldsymbol{N}_{\boldsymbol{o}}}\boldsymbol{)}$, that is $A_{o}\boldsymbol{e}_{\boldsymbol{i}}\boldsymbol{=}\boldsymbol{j}_{\boldsymbol{i}}$. With the linearity of the system, the output $\boldsymbol{E}_{\boldsymbol{o}}\boldsymbol{=}\boldsymbol{\Sigma}_{\boldsymbol{i}}\alpha_{i}\boldsymbol{e}_{\boldsymbol{i}}\boldsymbol{=}\left[ \boldsymbol{e}_{\boldsymbol{1}}\boldsymbol{,}\boldsymbol{e}_{\boldsymbol{2}}\boldsymbol{,\ldots,}\boldsymbol{e}_{\boldsymbol{N}_{\boldsymbol{j}}} \right]\left[ \alpha_{1},\alpha_{2},\ldots, \alpha_{N_{j}} \right]^{\boldsymbol{T}}$**,** which shows that the $\boldsymbol{E}_{\boldsymbol{o}}\boldsymbol{=}\left[ \boldsymbol{e}_{\boldsymbol{1}}\boldsymbol{,}\boldsymbol{e}_{\boldsymbol{2}}\boldsymbol{,\ldots,}\boldsymbol{e}_{\boldsymbol{N}_{\boldsymbol{j}}} \right]\boldsymbol{J}$**.** Comparing this with the governing forward Maxwell equation $A_{o}\boldsymbol{E}_{\boldsymbol{o}}\boldsymbol{=J}$**,** we conclude that $A_{o}^{+}=\left[ \boldsymbol{e}_{\boldsymbol{1}}\boldsymbol{,}\boldsymbol{e}_{\boldsymbol{2}}\boldsymbol{,\ldots,}\boldsymbol{e}_{\boldsymbol{N}_{\boldsymbol{j}}} \right]$ satisfying $A_{o}^{+}A_{o}=\boldsymbol{I}$**.**

Plugging $A_{o}^{+}A_{o}=\boldsymbol{I}$ into the Maxwell equations for the input and output fields, the input field $\boldsymbol{E}_{\boldsymbol{i}}$ and the output field $\boldsymbol{E}_{\boldsymbol{o}}$ can then be connected through**,**

$\boldsymbol{E}_{\boldsymbol{o}}\boldsymbol{=}A_{o}^{+}A_{i}\boldsymbol{E}_{\boldsymbol{i}}$, (S1)

where the $A_{o}^{+}$ and $A_{i}$ are parameterized with the $\epsilon_{r}$. We can observe from Eq. S1 that the linear propagation of the light field delineates a linear layer that connects the input and output with the full-connection weight matrix $W(\epsilon_{r})=A_{o}^{+}A_{i}$. We further consider the gradient-descent design of the parameters and the relay between layers. These starts with the observations that the manipulation of the wave field comes in two forms: modulation and coherent interference, the modulation can be formulated as the point-wise multiplication, and the coherent interference can be formulated as the addition in the complex field. Considering the modulation process: $\boldsymbol{E}_{\boldsymbol{i}}\boldsymbol{=}\boldsymbol{E}_{\boldsymbol{i}}\boldsymbol{'}exp(j\sqrt{\boldsymbol{\epsilon}_{\boldsymbol{r}}}(\Delta d)/\lambda)=\boldsymbol{E}_{\boldsymbol{i}}\boldsymbol{'}exp(j(\boldsymbol{n}_{\boldsymbol{R}}+i\boldsymbol{n}_{\boldsymbol{I}})(\Delta d)/\lambda)$, where $\Delta d$ is the modulation distance, $\lambda$ the wavelength, $\boldsymbol{n}_{\boldsymbol{R}}\boldsymbol{,}\boldsymbol{n}_{\boldsymbol{I}}$ the real and imaginary parts of refractive index. Incorporating the coherent reference beam, we have $\boldsymbol{E}_{\boldsymbol{i}}^{\boldsymbol{'}}\boldsymbol{=}\left( \boldsymbol{E}_{\boldsymbol{i}_{\boldsymbol{0}}}\boldsymbol{+}\boldsymbol{E}_{\boldsymbol{i}_{\boldsymbol{ref}}} \right)\boldsymbol{.}$ So the input field is expanded as $\boldsymbol{E}_{\boldsymbol{i}}\boldsymbol{=}\left( \boldsymbol{E}_{\boldsymbol{i}_{\boldsymbol{0}}}\boldsymbol{+}\boldsymbol{E}_{\boldsymbol{i}_{\boldsymbol{ref}}} \right)exp(2\pi j(\boldsymbol{n}_{\boldsymbol{R}}+i\boldsymbol{n}_{\boldsymbol{I}})(\Delta d)/\lambda)$. The end-to-end embedded neural network representations are

$\boldsymbol{y}\boldsymbol{=}f\left( \boldsymbol{x};n_{R,}n_{I},\boldsymbol{B} \right)=W\left( \boldsymbol{n}_{\boldsymbol{R}}^{\left( \boldsymbol{0} \right)}\boldsymbol{,}\boldsymbol{n}_{\boldsymbol{I}}^{\left( \boldsymbol{0} \right)} \right)(M\left( \boldsymbol{n}_{\boldsymbol{R}}^{\left( \boldsymbol{t} \right)}\boldsymbol{,}\boldsymbol{n}_{\boldsymbol{I}}^{\left( \boldsymbol{t} \right)} \right)\cdot\left( \boldsymbol{x+B} \right)\boldsymbol{)}$**,** (S2)

where $\boldsymbol{x}=\boldsymbol{E}_{\boldsymbol{i}_{\boldsymbol{0}}}$**,** $\boldsymbol{y}=\boldsymbol{E}_{\boldsymbol{o}}$**,** $M\boldsymbol{=}exp(2\pi j(\boldsymbol{n}_{\boldsymbol{R}}^{\left( \boldsymbol{t} \right)}\boldsymbol{+i}\boldsymbol{n}_{\boldsymbol{I}}^{\left( \boldsymbol{t} \right)})(\Delta d)/\lambda)$, and $\boldsymbol{B=}\boldsymbol{E}_{\boldsymbol{i}_{\boldsymbol{ref}}}$ are mapped to the input, output, complex multiplicative and biased weights in the neural network implementation. Here the $(\boldsymbol{n}_{\boldsymbol{R}}^{\left( \boldsymbol{0} \right)}\boldsymbol{,}\boldsymbol{n}_{\boldsymbol{I}}^{\left( \boldsymbol{0} \right)})$ and $(\boldsymbol{n}_{\boldsymbol{R}}^{\left( \boldsymbol{t} \right)}\boldsymbol{,}\boldsymbol{n}_{\boldsymbol{I}}^{\left( \boldsymbol{t} \right)})$ represents the refractive index in the fixed and tunable regions respectively. Note that we omit the reference input for simplicity in the main text. In real applications, the system is designed for the target *T*, and the associated quantitative loss function is represented as $L$. The gradient with reference to the complex refractive indexes $n=n_{R}^{\left( t \right)}+in_{I}^{\left( t \right)}$ are

$\frac{\partial L}{\partial\boldsymbol{n}}=-\frac{2\Delta d}{\lambda}conj\left( j\left( \boldsymbol{M}\cdot\left( \boldsymbol{x}+\boldsymbol{B} \right)\cdot{(W}^{T}\frac{\partial L}{\partial\boldsymbol{y}} \right) \right).$ (S3)

Also, the gradient of the input and reference systems are

$\frac{\partial L}{\partial\boldsymbol{x}}=\frac{\partial L}{\partial\boldsymbol{B}}=\boldsymbol{M}\cdot{(W}^{T}\frac{\partial L}{\partial y})$ (S4)

In this way, the gradients of all the parameters in the system can be retrieved and designed with the gradient descent. In the next section we derive fully-forward-mode gradient descent learning to self-design the embedded neural networks.

# Supplementary Note 2. Derivation of fully forward-mode gradient descent

**2.1 Gradient descent in optical system**

In this section, we formulate the gradient descent algorithm of a multi-layer parameterized optical system. Reformulate the propagation matrix $W$ in Supplementary Note 1 as the Green's function of the *k*-th linear optical layer as $G_{k}\left( r_{o}, r_{i} \right)$, the forward propagation can be formulated as

$y_{k}(r_{o})= \int G_{k}\left( r_{o}, r_{i} \right) x_{k}\left( r_{i} \right)d(r_{i})$. (S5)

The output can be nonlinearly activated and fed into the subsequent layers, $x_{k+1}=f(y_{k})$. The figure of merit is evaluated on the last output, $L=\Psi(y_{N}, T)$, where T is the target output. To efficiently evaluate the loss, the gradient is calculated with the loss function *L*. The gradient of $y_{N}$ is

$\delta y_{N}=\frac{\partial L}{\partial\boldsymbol{y}_{\boldsymbol{N}}}=\Psi_{x}^{'}\left( y_{N}, T \right).$ (S6)

From the chain rule, the gradient is propagated as

$\delta_{x_{k}}\left( r_{i} \right)=\int\delta_{y_{k}}\left( r_{o} \right)\frac{\partial y_{k}\left( r_{0} \right)}{\partial x_{k}\left( r_{i} \right)} d\left( r_{o} \right)=\int G_{k}\left( r_{o}, r_{i} \right) \delta_{y}\left( r_{o} \right) d(r_{o})$. (S7)

From Lorentz reciprocity, $G_{k}\left( r_{o}, r_{i} \right)=G_{k}\left( r_{i}, r_{o} \right)$, such that,

$\delta_{x_{k}}\left( r_{i} \right)=\int G_{k}\left( r_{i}, r_{o} \right) \delta_{y}\left( r_{o} \right) d(r_{o}).$ (S8)

which corresponds to propagating the field $\delta_{y}$ from the output of the k-th layer to the input of the *k*-th layer.

**2.2 Fully forward-mode gradient descent**

The complexity of backward propagating nature of the gradient algorithm in previous section inhibits training of general systems. To accommodate the free-space and integrated optical system, it would be ideal to train the optical system with only forward propagation. In this section, we prove that with spatial symmetry, data and error propagation can share the same forward path.

In a mirror symmetry system $\sigma_{v}$, we have $G\left( r_{i}, r_{o} \right)=G\left( \sigma_{v}(r_{i}), \sigma_{v}(r_{o}) \right)$. Let $(r_{o}^{'}, r_{i}')=(\sigma_{v}(r_{i}), {\sigma_{v}(r}_{o}))$, we then achieve a one-to-one match of $\left( r_{i}, r_{o} \right)$ with $(r_{o}^{'}, r_{i}')$, and $G\left( r_{i}, r_{o} \right)=G\left( r_{o}', r_{i}' \right)$. Consider the Eq. S8, for every $\left( r_{i}, r_{o} \right)$, there exists $(r_{o}^{'}, r_{i}')$ such that $G\left( r_{i}, r_{o} \right)=G\left( r_{o}', r_{i}' \right)$. Substituting $\left( r_{i}, r_{o} \right)$ in Eq. S8, we have $\delta_{x_{k}}\left( r_{o}' \right)=\int{d(r_{i}')G}_{k}\left( r_{o}', r_{i}' \right) \delta_{y}\left( r_{i}' \right)$, which corresponds to feeding the $\delta_{y}$ at the input of the system and forward-propagate it to the output.

In a rotation symmetry system $C_{Nv}$, suppose the input and output of the system has the relation of integer rotation symmetry, i.e., for any input $r_{i}\in\Omega_{in}$, there exists $r_{o}'=R(r_{i})\in\Omega_{out}$, and vice versa (for any point in the output $r_{o}\in\Omega_{out}$, there exist $r_{i}'=R^{-1}(r_{o})\in\Omega_{in}$ ). Let $(r_{o}', r_{i}')=(R(r_{i}), {R^{-1}(r}_{o}))$, when ${R^{-1}(r}_{o})= {R(r}_{o})$, we have $(r_{o}', r_{i}')=(R(r_{i}), {R(r}_{o}))$, such that $G\left( r_{i}, r_{o} \right)=G\left( r_{o}', r_{i}' \right)$. Substituting $\left( r_{i}, r_{o} \right)$ in Eq. S8, we again have $\delta_{x_{k}}\left( r_{o}' \right)=\int G_{k}\left( r_{o}', r_{i}' \right) \delta_{y}\left( r_{i}' \right) d(r_{i}')$, which corresponds to feeding the $\delta_{y}$ at the input of the system and forward-propagate it to the output. One important configuration is the equivalence ${R^{-1}(r}_{o})= {R(r}_{o})$, which requires the angle of rotation $R=\pi$ radian and to be integer number of symmetry rotation, that is, $\pi\equiv0 (\mathrm{mod}\frac{2\pi}{N})$. A sufficient condition is $N$ being an even number.

From section 2.1. and 2.2, we show that the gradient descent training can be realized with only forward propagation in the spatially-symmetrical reciprocal system.

# Supplementary Note 3. Spatial symmetries in free-space and integrated photonics

Spatial symmetry is inherent in optical systems. In free space optics, optical imaging and processing systems both entail symmetrical planes, where the optical path distance to and from this plane is symmetric. In photonic integrated circuits, the structure's topology can be engineered through nanofabrication to include spatially symmetrical patterns. In this section, we give an analysis of the spatial symmetries for the systems demonstrated in the main text.

**3.1 Free-space propagation**

From Rayleigh-Sommerfeld diffraction theory, the vectorized angular spectrum can be formulated as ^1^,

$U_{0}\left( f_{x}, f_{y} \right)\mathcal{=\mathcal{F}}\left( U\left( x, y \right) \right)$, (S9)

where $U\left( x, y \right), U_{0}\left( x, y \right),$ and $\mathcal{\mathcal{F}}$, represent the input vector, the output vector, and the Fourier transform respectively.

The propagation of the angular spectrum is

$U_{Z}\left( f_{x}, f_{y} \right)=D\left( e^{\frac{j2\pi}{\lambda}\sqrt{1-\alpha^{2}-\beta^{2}}z} \right)U_{0}\left( f_{x}, f_{y} \right)$ (S10)

The output light field reads,

$U_{z}\left( x, y \right)={\mathcal{\mathcal{F}}}^{-1}\left( U_{Z}\left( f_{x}, f_{y} \right) \right)$ (S11)

And the total transform now reads,

$U_{z}\left( x, y \right)={\mathcal{\mathcal{F}}}^{-1} D\left( e^{\frac{j2\pi}{\lambda}\sqrt{1-\alpha^{2}-\beta^{2}}z} \right)\mathcal{\mathcal{F}}U\left( x, y \right)$ (S12)

Denote $T(z)={\mathcal{\mathcal{F}}}^{-1} D\left( e^{\frac{j2\pi}{\lambda}\sqrt{1-\alpha^{2}-\beta^{2}}z} \right)\mathcal{\mathcal{F}}$, as the Fourier transform $\mathcal{\mathcal{F}}$, diagonal matrix $D\left( e^{\frac{j2\pi}{\lambda}\sqrt{1-\alpha^{2}-\beta^{2}}z} \right)$, and inverse Fourier transform ${\mathcal{\mathcal{F}}}^{-1}$are both symmetric, the transform $T$ is thus symmetric.

**Focusing through scattering media**

The spatial transformation in the setups for focusing through scattering media can be summarized as follows:

$S={T(z)D}_{sc}T(z)$ (S13)

The transformation is described as $T(z)$, where the term on the left- and right-hand side represents the free-space propagation to and from the scattering media, respectively. $D_{sc}$ denotes the scattering. In this case, since the input and output ports of the scattering matrix coincide, the matrix becomes symmetric. Consequently, the transformation itself possesses spatial symmetry.

**Non-line-of-sight imaging and processing**

The non-line-of-sight system is slightly more intricate, and the spatial transformation of the system can be summarized as follows:

$N= {T\left( z_{1} \right)R}_{ref}T\left( z_{2} \right)R_{ob}T\left( z_{2} \right)R_{ref}T(z_{1})$ (S14)

The propagation from the incident light to the wall is represented by $T\left( z_{1} \right)$ while the propagation from the diffusive reflector to the object is denoted by $T\left( z_{2} \right)$. $R_{ref}$ and $R_{ob}$ represent the transformations of the optical field when the light reflects from the wall and the object, respectively. Despite the complexity, it is noteworthy that the transformation can still be described by a symmetrical matrix, indicating symmetry of the system.

**3.2 Integrated photonic systems**

The spatial symmetry is ubiquitous in integrated systems. Symmetry has provided ease to analyze the micro photonic systems, for example in the photonic crystals and meta-surfaces ^2, 3^. The holes and slots are designed with symmetries to control the photonic band structures.

In the PIC neural network, we constructed integrated photonic symmetry with tunable photonic mesh. The symmetry was configured by tuning the attenuation coefficient of the modulators in the circuit grids. The programming fidelity is shown in Fig. 5c of the main text. The standard deviation of the temporal drifting of the symmetry matrix values were only 0.012%, 0.012%, and 0.010%.

For the evaluation of non-Hermitian band braiding, a coupled waveguide consisting two waveguides was used. The gain/loss of the waveguide was set to be uniform along the propagation, which can be realized by uniform doping and pumping for gain and loss, respectively. As a result, the wave propagation from the input to the output is the same as that of the wave propagation from the output to the input, so the symmetry condition holds.

# Supplementary Note 4. Validation of spatial symmetric reciprocity via two-step propagation

To validate the spatial symmetry and the accuracy of complex fields measurement, we employed the method of spatially symmetric conjugation (Fig. S21a). In Supplementary Note 1, we had the transmission function as $\boldsymbol{y}=WM\boldsymbol{(x+B)}$**.** Here we analyze the propagation $\boldsymbol{y}=W\boldsymbol{x}$**.** When the system is unitary and symmetry, $W$ will satisfy $WW^{*}=I$, where $I$ is the identity matrix and * represents the conjugation operation. Propagating through the system twice with the conjugation of the first output as the input of the second propagation, we will have

$\boldsymbol{y}_{\boldsymbol{2}}\boldsymbol{=}W\boldsymbol{y}_{\boldsymbol{1}}\boldsymbol{=}W\left( W\boldsymbol{x} \right)^{\boldsymbol{*}}\boldsymbol{=}\boldsymbol{x}^{\boldsymbol{*}}$**.**  (S15)

In this way, we can recover the amplitude of the original inputs with twice propagation under the symmetric and unitary constraints, which means when we recapture the original inputs at the second-round output, we can validate the symmetry of our system.

However, unitary is a strict constraint facilitated by the losslessness of the system. In more generic cases, the system may not always be unitary. For example, light may be scattered sideways, or blocked in the transversal direction. In this case, we represent the propagation matrix as $W=U(I-H)$, with $U$ the unitary matrix and *H* the normalized difference between the $W$ and $U$. $\boldsymbol{y}_{\boldsymbol{2}}$ now reads $(I-H-UH^{*}U^{*}+HUH^{*}U^{*})\boldsymbol{x}^{\boldsymbol{*}}$**.** Denote $\alpha$ the eigen value of *H* with the largest amplitude, we have the L2-norm reading,

$\left| \left| \boldsymbol{y}_{\boldsymbol{2}}\boldsymbol{-}\boldsymbol{x}^{\boldsymbol{*}} \right| \right|\boldsymbol{=}\left| \left| Hx^{*}+UH^{*}U^{*}x^{*}-HUH^{*}U^{*}x^{*} \right| \right|\boldsymbol{\leq}3\left| \alpha\right| |\left| x^{*} \right||$ (S16)

It shows that the output similarity is upper bounded by $3\left| \alpha\right|$. As a result, we can still use the similarity between the twice propagated output and input as metric to validate the symmetry of the optics even under the non-unitary condition. In a single measurement of a complex field, if the result obtained from the conjugate reverse output is close to the theoretical value, it indicates high level of symmetry.

To test the above development method, before FFM learning experiment, we project the system with an input $\boldsymbol{x=}{\boldsymbol{A}_{\boldsymbol{I}}e}^{j\boldsymbol{\phi}_{\boldsymbol{I}}}$, after propagating through the system, we obtain a complex field of $\boldsymbol{y}_{\boldsymbol{1}}={\boldsymbol{A}_{\boldsymbol{O}}e}^{j\boldsymbol{\phi}_{\boldsymbol{O}}}$. The output $\boldsymbol{y}_{\boldsymbol{1}}$ is then phase conjugated and projected into the system again. The results are shown in Fig. S21b. In all three conditions, that is the scatter-free, the scatterer-I (level 4) and scatterer-II, we project the image "9" into the system and measure the output $\boldsymbol{y}_{\boldsymbol{1}}$. After conjugation, the $\boldsymbol{y}_{\boldsymbol{1}}$ is projected into the system again to obtain the output $\boldsymbol{y}_{\boldsymbol{2}}$. Even though the three different types of propagation media have quite distinct outputs $\boldsymbol{y}_{\boldsymbol{1}}$ (second row of Fig. S21b), the output of the second forward propagation $\boldsymbol{y}_{\boldsymbol{2}}$ all successfully recover the original inputs. We also observe that the scattering free media produces the best reconstruction, as the transportation matrix is closest to the unitary matrix, which corresponds with previously developed theory.

# Supplementary Note 5. FFM learning protocol

We utilize the FFMLearning function to achieve onsite machine learning (Algorithm 4). This function involves forward data propagation and complex field measurement performed by the ForwardData (Algorithm 1), and forward error propagation and complex field measurement executed by the ForwardError (Algorithm 2). Using the measured fields obtained from algorithms 1&2, the gradient computation is finally carried out using the FFMGradient function (Algorithm 3). The specific workflow of these functions is as follows.

**Algorithm 1** ForwardData: data propagation

**mainFunction** ForwardDataBatch (*X, M*)

**input** data: $X\in C^{L\times S\times S}$

reconfigurable modulation parameters: $M=\left[ M_{1},M_{2},\ldots,M_{N} \right] (M_{k}=exp(2\pi j\left( n_{R-k}+1jn_{I-k} \right)\Delta d/\lambda), n_{R-k},n_{I-k}\in R^{S\times S},k=1,2,\ldots,N)$

**Output** output field: $U\in C^{L\times S\times S}$

complex field of data propagation for gradient calculation: $P_{d}\in C^{L\times S\times S}$

gradient of the nonlinear activation: $P_{g}\in C^{L\times N\times S\times S}$

$x_{1},x_{2},\ldots,x_{L}\leftarrow X$

**for** $l\in\left[ 1,2,\ldots,L \right]$ **do**

$U_{l},{P_{d}}_{l}, P_{g_{l}}\leftarrow$ForwardData($x_{l},M$)

**end for**

$U\leftarrow[U_{1},U_{2},\ldots,U_{L}]$

$P_{d}\leftarrow[{P_{d}}_{1},{P_{d}}_{2},\ldots,{P_{d}}_{L}]$

$$P_{g}\leftarrow[{P_{g}}_{1},{P_{g}}_{2},\ldots,{P_{g}}_{L}]$$

**return** $U,P_{d},P_{g}$

**end function**

**subFunction1** ForwardData (*x, M*)

**input** data: $x\in C^{S\times S}$

reconfigurable modulation parameters: $M=\left[ M_{1},M_{2},\ldots,M_{N} \right] (M_{k}=exp(2\pi j\left( n_{R-k}+1jn_{I-k} \right)\Delta d/\lambda), n_{R-k},n_{I-k}\in R^{S\times S},k=1,2,\ldots,N)$

**Output** output field: $U_{No}\in C^{S\times S}$

complex field of data propagation for gradient calculation: ${Pk}_{d}\in C^{N\times S\times S}$

gradient of the nonlinear activation: ${Pk}_{g}\in C^{N\times S\times S}$

**for** $k\in\left[ 1,2,\ldots,N \right]$ **do**

${{Pk}_{d}}_{k}\leftarrow M_{k}x$

$x\leftarrow M_{k}x$

$x\leftarrow SystemForward(x)$

$${Pk}_{g_{k}}\leftarrow f'(x)$$

$x\leftarrow f(x)$

**end for**

$U_{No}\leftarrow x$

${Pk}_{d}\leftarrow\left[ {{Pk}_{d}}_{1},{{Pk}_{d}}_{2},\ldots,{{Pk}_{d}}_{N} \right]$

$${Pk}_{g}\leftarrow\left[ {Pk}_{g_{1}},{Pk}_{g_{2}},\ldots,{Pk}_{g_{N}} \right]$$

**return** $U_{No},{Pk}_{d},{Pk}_{g}$

**end function**

**subFunction2** $SystemForward(x)$

**input** data: $x\in C^{S\times S}$

**Output** output field: *output*$\in C^{S\times S}$

$output\leftarrow x travels forward through system$.

**return** *output*

**end function**

Notations:

*S*: pixel number of modulated region

*N*: layer number

*L*: sample number of a batch

$f$: nonlinear activation function

**Algorithm 2** ForwardError: error propagation

**mainFunction** ForwardErrorBatch (*E, M,*$P_{g}$)

**input** error: $E\in C^{L\times S\times S}$

reconfigurable modulation parameters: $M=\left[ M_{1},M_{2},\ldots,M_{N} \right] (M_{k}=exp(2\pi j\left( n_{R-k}+1jn_{I-k} \right)\Delta d/\lambda), n_{R-k},n_{I-k}\in R^{S\times S},k=1,2,\ldots,N)$

gradient of the nonlinear activation: $P_{g}\in C^{L\times N\times S\times S}$

**Output** complex field of error propagation for gradient calculation: ${Pk}_{e}\in C^{L\times N\times S\times S}$

$e_{1},e_{2},\ldots,e_{L}\leftarrow E$

$$P_{g_{1}},P_{g_{2}},P_{g_{3}}, \ldots P_{g_{L}}\leftarrow P_{g}$$

**for** $l\in\left[ 1,2,\ldots,L \right]$ **do**

${P_{e}}_{l}\leftarrow$ForwardError ($e_{l},M,P_{g_{l}}$)

**end for**

$P_{e}\leftarrow{P_{e}}_{1},{P_{e}}_{2},\ldots,{P_{e}}_{L}$

**return** $P_{e}$

**end function**

**subFunction1** ForwardError (*e, M,* ${Pk}_{g}$)

**input** error: $e\in C^{S\times S}$

reconfigurable phase masks: $M=\left[ M_{1},M_{2},\ldots,M_{N} \right] (M_{k}=exp(2\pi j\left( n_{R-k}+1jn_{I-k} \right)\Delta d/\lambda), n_{R-k},n_{I-k}\in R^{S\times S},k=1,2,\ldots,N)$

gradient of the nonlinear activation: ${Pk}_{g}\in C^{N\times S\times S}$

**Output** complex field of error propagation for gradient calculation: ${Pk}_{e}\in C^{S\times S}$

${Pk}_{g_{1}},{Pk}_{g_{2}},{Pk}_{g_{3}}, \ldots{Pk}_{g_{N}}\leftarrow{Pk}_{g}$

**for** $k\in\left[ N,N-1,\ldots,1 \right]$ **do**

$e\leftarrow{Pk}_{g_{k}}e$

$e\leftarrow SystemForward(e)$

${{Pk}_{e}}_{k}\leftarrow e$

$e\leftarrow M_{k}e$

**end for**

${Pk}_{e}\leftarrow\left[ {{Pk}_{e}}_{1},{{Pk}_{e}}_{2},\ldots,{{Pk}_{e}}_{N} \right]$

**return** ${Pk}_{e}$

**end function**

**subFunction2** $SystemForward(x)$

**input** data: $x\in C^{S\times S}$

**Output** output field: *output*$\in C^{S\times S}$

$output\leftarrow x travels forward trough system$.

**return** *output*

**end function**

Notations:

*S*: pixel number of modulated region

*N*: layer number

*L*: sample number of a batch

**Algorithm 3** FFMGradient: gradient calculation

**mainFunction** FFMGradient (*X, T, M*)

**input** data: $X\in C^{L\times S\times S}$

target: *T* $\in R^{L}$

reconfigurable phase masks: $M=\left[ M_{1},M_{2},\ldots,M_{N} \right] (M_{k}=exp(2\pi j\left( n_{R-k}+1jn_{I-k} \right)\Delta d/\lambda), n_{R-k},n_{I-k}\in R^{S\times S},k=1,2,\ldots,N)$

**Output** gradient: $g\in R^{N\times S\times S}$

$U,P_{d},P_{g}$= ForwardDataBatch (*X, M*)

$Y_{o}\leftarrow\left| U \right|^{2}$

*Loss*$\leftarrow\left| \left| Y_{o}-T \right| \right|^{2}$

$E\leftarrow conj\left( U \right)*\partial Loss/\partial Y_{o}$

$P_{e}$= ForwardErrorBatch (*E, M,*$P_{g}$)

$g \leftarrow$GradienBatch ($P_{d},P_{e}$)

return $g$

**end function**

**subFunction1** GradientBatch ($P_{d},P_{e}$)

**input** complex field of data propagation for gradient calculation: $P_{d}\in C^{L\times S\times S}$

complex field of error propagation for gradient calculation: $P_{e}\in C^{L\times S\times S}$

**Output** gradient: $g\in R^{N\times S\times S}$

${{Pk}_{d}}_{1},{{Pk}_{d}}_{2},\ldots,{{Pk}_{d}}_{L}\leftarrow P_{d}$

${{Pk}_{e}}_{1},{{Pk}_{e}}_{2},\ldots,{{Pk}_{e}}_{L}\leftarrow P_{e}$

**for** $l\in\left[ 1,2,\ldots,L \right]$ **do**

$\left[ {{Pk}_{d}}_{l1},{{Pk}_{d}}_{l2},\ldots,{{Pk}_{d}}_{lN} \right]\leftarrow{{Pk}_{d}}_{l}$

$\left[ {{Pk}_{e}}_{l1},{{Pk}_{e}}_{l2},\ldots,{{Pk}_{e}}_{lN} \right]\leftarrow{{Pk}_{e}}_{l}$

**for** $k\in\left[ 1,2,\ldots,N \right]$ **do**

$g_{lk} \leftarrow-conj(i{{Pk}_{d}}_{lk}*{{Pk}_{e}}_{lk})$

**end for**

**end for**

**for** $k\in\left[ 1,2,\ldots,N \right]$ **do**

$g_{k} \leftarrow mean(g_{1k},g_{2k},\ldots,g_{Lk})$

**end for**

$g\leftarrow[g_{1},g_{2},\ldots,g_{N}]$

**return** $g$

**end function**

Notations:

*S*: pixel number of modulated region

*N*: layer number

*L*: sample number of a batch

**Algorithm 4** FFMLearning

**mainFunction** FFMLearning (*X, T, M*)

**input** data: $X\in C^{L_{all}\times S\times S}$

target: *T* $\in R^{L_{all}}$

reconfigurable phase masks: $M=\left[ M_{1},M_{2},\ldots,M_{N} \right]$

$(M_{k}\in\left[ 0,2\pi\right]^{S\times S},k=1,2,\ldots,N)$

**Output** none

$h\leftarrow0$

**for** $b\in\left[ 1,2,\ldots,B \right]$ **do**

random sample $X_{b},T_{b}$ from $X,T$.

**require** $X_{b}\in C^{L\times S\times S}$

**require** $T_{b}\in R^{L}$

$g_{b} \leftarrow FFMGradient(X_{b}, T_{b}, M)$

$\delta\eta,h\leftarrow Optimizer(g_{b}, h)$

$$\left( n_{R}+1jn_{I} \right)\leftarrow\left( n_{R}+1jn_{I} \right)-\delta\eta,h$$

**end for**

**end function**

Notations:

*S*: pixel number of modulated region

*N*: layer number

*L*: sample number of a batch

$L_{all}$: total sample number

*h*: gradient history

*B*: batch number

# Supplementary Note 6. PIC chip packaging

Two eight-channel fiber arrays with 127-µm period and 8° tilting angle were used for guiding the light to and from the photonic chip. After aligning the fiber arrays with the grating arrays under the microscope with the aid of nanometer motorized translation stage, the vertically coupled fiber arrays were attached and fixed to the PIC with curable epoxy. The insertion losses of the fiber-to-chip coupling after packaging is measured around -8 dB per channel.

A two-layer dedicated PCB board was developed for on-chip electrical signal feeding. The on-chip bond pads of VOA array with 100-µm period were connected to the PCB board by gold wire bonding and independently routed to electrical socket with signal wires of 800-µm period. A customized multi-channel DC signal source connects to the board and controls the injection current of VOA by setting the voltage to be 0-5V, which effectively tuned the imaginary parts of the effective refractive of the guided mode wave.

For thermal stabilization, the die core and a thermistor were securely mounted on a copper block using thermal adhesive. The thermistor measured the temperature of the chip, and a thermal-electrical temperature controller (TEC, Peltier cooler) was attached to the copper block to cool the packaged system. A proportional-integral-derivative (PID) feedback loop was established between the thermistor and the TEC. This feedback loop enables maintenance of the on-chip temperature within ± 0.004 Kelvin (corresponding to a ± 2 Ω long-term thermistor resistance stability), ensuring reliable and consistent operation.

# Supplementary Note 7. Methods for comparison

**7.1 Electronic artificial neural network (Fig. S22)**

The neural network used for comparison in classification tasks was a modified version of ResNet-18 ^4^. The network begins with a convolutional layer followed by a max pooling operation which transforms the 1-channel input into a 64-channel data. The subsequent stages, referred to as Stage 1 to Stage 4, each consist of a set of residual blocks. These blocks are responsible for capturing increasingly complex and abstract features as information flows through the network. The number of blocks in each stage varies, with Stage 1 having two blocks while Stages 2,3,4 all having 2 blocks. Within each residual block, there are typically two or three convolutional layers with batch normalization, rectified linear unit (ReLU) activation functions. To reduce the dimensionality of feature maps, 1×1 convolutional layers are utilized within the residual blocks, acting as bottleneck layers. The final layers of the ResNet-18 architecture include global average pooling, which aggregates the spatial features into a vector, and a fully connected layer followed by a SoftMax activation function for classification.

The neural network we used for comparison in imaging tasks was a U-Net ^5^. The U-Net architecture consists of a contracting path (left side) and an expansive path (right side). The contracting path follows the standard convolutional network architecture. It involves repeated application of two 3×3 convolutions (without padding), followed by a rectified linear unit (ReLU) and a 2×2 max pooling operation with a stride of 2 for down-sampling. With each down-sampling step, the number of feature channels is doubled. The expansive path is responsible for up-sampling the feature map. It consists of an up-sampling operation followed by a 2×2 convolution (referred to as "up-convolution") that reduces the number of feature channels by half. The up-sampled feature map is then concatenated with the corresponding cropped feature map from the contracting path. After concatenation, two 3×3 convolutions are applied, each followed by a ReLU activation. Cropping is necessary to compensate for the loss of border pixels during each convolution operation. The final layer of the U-Net utilizes a 1×1 convolution to map each 64-component feature vector to the desired number of classes. The entire network comprises a total of 23 convolutional layers.

**7.2 Optimization methods for comparison**

Here we briefly discuss the simulated annealing ^6^ and PSO optimization ^7^ used for comparison.

Simulated annealing is a metaheuristic optimization algorithm inspired by the annealing process in metallurgy. It explores the solution space by iteratively making random changes to the current solution and evaluating their quality. The algorithm accepts inferior solutions based on a probabilistic criterion, allowing it to escape local optima. The acceptance probability is controlled by a temperature parameter, which gradually decreases over time. Initially, a high temperature enables exploration, while a lower temperature promotes exploitation. This balance between exploration and exploitation helps simulated annealing converge towards an optimal or near-optimal solution for combinatorial optimization problems. The start temperature and the annealing rate were tuned and set to 1.0 and 0.97 respectively.

Particle swarm optimization (PSO) is a population-based optimization algorithm that simulates the collective behavior of a swarm of particles. Each particle represents a potential solution in the search space. The particles move through the search space, adjusting their positions based on their own best-known position and the best-known positions of neighboring particles. The movement is guided by two factors: the cognitive component, which represents the particle's memory of its own best solution, and the social component, which represents the particle's knowledge of the best solution found by any particle in its neighborhood. Through iterative updates, the particles dynamically explore the search space, converging towards promising regions and gradually improving the overall solution quality. During the experiment, the population, the inertia, the cognitive weight, and the social weight were tuned and set to 10, 0.5, 0.5, 0.5 respectively.

Simulated annealing and PSO optimization are used for constructing comparison in imaging through scatter and NLOS tasks. We employed a set of first six Zernike polynomial coefficients as basis for generating the phase parameters in the design space. Specifically, the phase in the design space is equal to the inner product of a set of trainable coefficients and this basis set. These trainable coefficients serve as the search targets for the optimization algorithm. The mean square error loss between the resulting image and the ground truth was used as the loss function. With this experimental setup, we optimized the phase to achieve the imaging task.

# Supplementary Note 8. Model-free non-Hermitian topological band braiding in the photonic integrated circuits

Conventionally, the analysis of non-Hermitian system starts with fully understood model of Hamiltonian. Here we set out to discover underlying relation in a model-free way by controlling the behaviors of the system with the FFM learning method. For the ease of illustration, we layout the problem in a forward manner. Consider the coupled system governed by non-Hermitian Hamiltonian,

$\boldsymbol{H}=\kappa\boldsymbol{a}^{\boldsymbol{\dagger}}\boldsymbol{b}+\kappa\boldsymbol{b}^{\boldsymbol{\dagger}}\boldsymbol{a+}\frac{i\gamma_{G}}{2}\boldsymbol{a}^{\boldsymbol{\dagger}}\boldsymbol{a-}\frac{i\gamma_{L}}{2}\boldsymbol{b}^{\boldsymbol{\dagger}}\boldsymbol{b}$, (S17)

where $\boldsymbol{a}^{\boldsymbol{\dagger}}\boldsymbol{(a),}$ and $\boldsymbol{b}^{\boldsymbol{\dagger}}\boldsymbol{(b)}$ are the creation (annihilation) operators for the first and second modes of the system, and $\kappa, \gamma_{G},\gamma_{L}$ are the coupling coefficient, gain, and loss respectively.

Without solving the Hamiltonian, we constructed a spatially coupled single waveguide system with tunable gain/loss, while keeping the coupling coefficient fixed (Extended Data Fig. 1a). It can be observed that injecting an arbitrary amplitude into the waveguide would result in two spatially varying field in the waveguide. The field distribution across the two channels is symmetric and periodic (Extended Data Fig. 1c, PT-symmetric region). Here we guide the spatially reciprocal distribution to evolve to non-reciprocal outputs. Specifically, we select maximization or minimization of the output from the first waveguide as our target. The loss function to be minimized reads,

$L = \left| \left| y_{a}-T \right| \right|.$ (S18)

The cornerstone facilitating the FFM learning in this problem is the symmetry of the refractive index in the propagation direction. This requires the gain and loss in the first and second waveguide to be constant, which can be achieved by uniform doping and pumping for loss and gain, respectively. Similar to the setup shown in Fig. 2a of main text, the output phase and amplitude can be measured with cameras and an interference beam of light. In the forward and error propagation, the phase and amplitude of inputs are configured with phase and amplitude modulators. During the simulation, we selected the normalized coefficient of $\kappa,$and propagation distance to be 1.9, and 0.03 respectively. And the $\gamma_{G}=\gamma_{L}=\gamma$ was the tunable variable dependent on the pumping power.

The resulting evolution of the output intensity is shown in Extended Data Fig. 1b, with typical field distributions at certain steps shown on the right panel. From 0 to 200 ^th^ iteration, the target $T$ is set to 200, which correspond to maximization of the output. From 200^th^ to 300^th^ iteration, the target is set to 8, which effectively minimizes the output. It can be shown that the system was guided to reversibly switch from the reciprocal to non-reciprocal region, and vice versa. The turning point is $\gamma=2\kappa$ (Extended Data Fig. 1c).

We then solved the Hamiltonian represented by Eq. S17 and visualize the complex band diagram in Extended Data Fig. 1d. It is shown that the switching point realized from the FFM learning is the branching point of the energy diagram, that is, the exceptional point (EP). And the process of the learning evolution in Extended Data Fig. 1b actually correspond to the non-Hermitian braiding group 𝔹_2_ (Extended Data Fig. 1e). In a self-design way, we search out the exceptional point and achieve band braiding.

# Supplementary Note 9. Summary of the FFM learning

The results are summarized in Table S1. **Free-space systems**. Because of the inherently embodied physical system of the FFM learning, each propagation takes same amount of time, regardless of the system complexity. In the demonstrated free-space applications (the deep neural networks, the focusing through scattering media, the parallel NLOS imaging, and the all-optical NLOS processing), commercial spatial light modulators could have a speed of over 1 kiloHertz (HSP1K-488-800-PC8, Meadowlark Optics), thus the propagation time is **less than 2.0 ms** per iteration. The performances of the designed systems are specifically, **1.** **In deep free-space neural network**, the FFM onsite design converged to 92.5% accuracy with 3000 iterations of training on Fashion-MNIST dataset. The in-silico training on standard GPU accelerators also took ~1.78 ms per iteration and converged after 3000 iterations of training, which were on the same level of FFM learning. However, because of the mismatch and accumulated errors of deep neural networks, the deployed in-silico system only had 58.8% testing accuracy. With further incorporation of nonlinear activation function, four-layer FFM achieved 93.0%, 60.0%, and 59.5% on MNIST, CIFAR, and ImageNet sub-datasets. **2. For focusing through scattering media**, the FFM learning achieves average FWHM of 81.2 µm within 25 iterations. On the other hand, each iteration of the optimization-based learning method takes ~1.0 ms, as the optimization entails only data propagation, while achieving 120.0-µm FWHM focal points on average after 400 iterations of optimization. **3.** **The NLOS amplitude imaging task** learn the pixelated imaging of objects grids. The FFM learning of 3×3 objects grid took 100 iterations with 1.0-SSIM imaging quality, while the optimization methods took 500 iterations with 0.36-SSIM imaging quality. **4.** **In NLOS phase imaging task**, FFM learning of imaging and processing took 20 and 6000 iterations to converge, respectively, while the state-of-the-art ANN took 25 and 6000 iterations. Under ambient noise, the ANN-based architecture deteriorated severely, with 15 dB, 13 dB, and 11 dB SNR level, the imaging quality of ANN-based methods degraded to SSIM indices of 0.40, 0.35, and 0.31, compared with 0.81, 0.79, and 0.75 SSIM indices of the FFM learning. **5. For NLOS all-optical processing,** The FFM learning requires less than 1 photon to achieve 94.5% binary classification accuracy, while ANN-based methods require 37.6 photons to reach the same performances. The FFM learning also learns with fewer training data, and thus have more robust performances with the diffusive reflector rotating at 2.5 degrees per learning iteration. **Integrated photonic systems.** In integrated photonic neural network, FFM learning achieves iteration time of 64 ms with micro-electromechanical systems (MEMS) VOA of 1 kHz bandwidth. Comparatively, each iteration of the in-silicon training is 2-ms. However, the in-silico trained network degrades to 71.7% accuracy after being deployed to the physical system, far below 94.2% of the FFM learning results.

We have demonstrated that the FFM learning results have better performances than in-silico learned system and onsite optimization, and orders of magnitude faster speed than the other optimization-based designed methods. The prototype experimental results have similar speed with the GPU accelerated designed methods. To further accelerate the implementation, faster spatial light modulators have been predicted by the research community^8^, and higher speed of integrated photonic modulators are also available, which will reduce the propagation time to less than 1 ns^9, 10^. So the iteration time of integrated photonic neural network learning can be reduced to 64 ns by using Mach-Zehnder modulator which is almost 30,000 times faster than 1ms per iteration of in-silico learning method.

# Supplementary Note 10. FFM and other emerging neural network training methods

Recent advancements in optics and electronics have resulted in notable developments in neural network training. Here we discuss the other two representative methods, that is, the in-situ backpropagation training^11^, and forward-forward algorithm^12^.

The work of in-situ training implements neural network training by physically propagating the light field backwards through a neural network for the error propagation in an on-chip 4×4 MZI unitary mesh. Error backpropagation is a widely-used approach in training artificial neural network on electronic computers. However, in optics, backpropagating light with descent alignment accuracy to the forward beam is not trivial work, which may be an obstacle for scaling up the backpropagation methods. Also, as this backpropagation protocol reckons on the unitarity of the forward propagation, the method has only been demonstrated on unitary MZI mesh, whether the method can be generalized to non-unitary scenario remains to be explored.

Unlike the discussed method that strictly follows the electronic ANN training approach, the proposed FFM method employs innate symmetry of the optics, which alleviates the backpropagated light and thus facilitates learning of more general systems. In this work, we demonstrate training of million-parameter free-space neural networks (160,000 parameters per layer) and multi-layer on-chip optical neural network. More than that, we demonstrate that the FFM can broaden its boundary beyond optical neural network training to enable high-performance imaging, photon-efficient all-optical processing, and self-learning physics.

In electronic ANN, a forward-forward algorithm is recently revealed to alleviate the backpropagation. [The forward-forward algorithm: Some preliminary investigations. *arXiv preprint arXiv:2212.13345*.]. However, there may be some limitations of FFA when deployed onto optical systems.

The reasons are that, even though the FFA method alleviates the backpropagation between layers, it still needs to compute derivatives within one layer. In optical neural networks, the parameters are in essence the effective refractive index of the tunable materials, and directly applying FFA to optical neural networks to take derivatives with respect to the optical parameters would still entail backpropagating the light field from the output of one layer to where the parameters are located^11, 13, 14^, or it should involve offline calculation of matrix-vector operations^15^, which means that FFA does not completely solve the BP problems in optics.

Other limitations are that at current stage, the FFA may not generalize as well as other explicit end-to-end gradient descent methods, as noted by the author in the paper:

*The forward-forward algorithm is somewhat slower than backpropagation and does not generalize quite as well on several of the toy problems investigated in this paper so it is unlikely to replace backpropagation for applications where power is not an issue. The exciting exploration of the abilities of very large models trained on very large datasets will continue to use backpropagation.*

To summarize, realizing a fully onsite learning platform with FFA is still challenging.

The advantage of the FFM learning in neural network training is that, both propagation processes, the data propagation and error propagation are deployed on the optical system. Considering an optical layer with *M* inputs and *M* outputs, the number of computes in the data and error propagations are ~$O(M^{2})$, while the number of operations connecting adjacent layers is ~$O(M)$. As a result, a proportion of ~$\left( \frac{M}{M+1} \right)$ operations are implemented in the optical domain and takes full advantage of the parallelism of the optics. As the FFM alleviates the need of ultraprecise alignment between the forward and backward light, the scale of $M$ can be as large as 10,000 to 1000,000 on state-of-the-art ONNs supported by large-scale modulator array (160,000 in our demonstrated experiments). So that majority of the operations are deployed on the optical computing.

# Supplementary Note 11. Parallel multilayer implementation of FFM learning and incorporation of nonlinear activation

In Fig. S4a, the propose of the full system of the multilayer FFM learning is provided. Each layer of the system consists of a 4*f*-conjugated amplitude and phase modulator. Part of the output light is split out for detection and the rest is fed into the next layer. In the error propagation, a flip mirror guides the laser into the system as the coherent source and the error propagation is locally performed at this layer. Fig. S4b illustrates the streamline of the multilayer FFM learning. During the data propagation, all the layers are parallelly recorded, which takes BS time steps (where BS stands for the batch size) to implement BS×N optical layers. All the layers work in full time. In the error propagation, considering a feedforward neural network architecture, the data can be streamlined as illustrated in Fig. S4(b). This involves propagating the first sample at the first timestep, then the first sample and the second sample at the second timestep, …. In this way, it takes (BS+N-1) timesteps for N-layer neural network by propagating in batch. On average, each layer of the optical system operates for BS/(BS+N-1) of the total time. For the more complex neural network architecture like ResNet^4^ and GPT-3^16^, assuming *L* parallel optical layers per network layer, the data and error propagation demand BS and (BS+N-1) timesteps for BS×N×L optical layers, respectively. The workload again averages to BS/(BS+N-1). We calculate the workload of typical neural network architectures in Table S2, encompassing the 8-layer ONN of this work, the 56-layer residual neural network^4^, and the large language model GPT-3^16^. Consequently, in these AI architectures, the workload of the error propagation ranges from 58.8% to nearly 100%. The time consumption is the inverse of the workload, (BS+N-1)/BS, indicating the error propagation consume only 1~1.70 times that of the data propagation. The error propagation can thus attain similar parallelism to the data propagation.

For the incorporation of the nonlinearity, we propose two versions of the nonlinearities for the data propagation. **Optoelectronic nonlinearity.** The data propagation process employs the photodetection and use the converted electronic signal to modify the optical propagation. **All-optical nonlinearity.** The all-optical nonlinearity realizes the nonlinear activation function completely in the optical domain, such as photorefractive effect, saturable absorption, etc.^17^. The system of the data propagation of the optoelectronic and all-optical nonlinearity can both be realized as described in Fig. S5a, where the output of data propagation in each layer is partially split out for recording and the rest of the light is nonlinearly activated before being propagated to the next layer. The gradient of the nonlinear function will be calculated based on the measured output. In the error propagation (Fig. S5b), the input error field will be multiplied by the pre-calculated gradient function and propagated to the output. Even though the gradient of the nonlinear activation is computed offline, it has only ~$O(M)$ operations. Considering that the data and error propagations of each layer contain ~$O(M^{2})$ operations, majority of the computes is implemented on the optical end on site. Also, because the nonlinear activations are calculated based on the measurements, the nonlinear FFM learning would not be limited by the mathematical form of the nonlinearity and be applicable to general functions.

To experimentally evaluate the effect of nonlinearity, we conducted experimental evaluation on diverse datasets. In addition to the propagation of each layer on the experimental system, we employed an optoelectronic nonlinear function digitally, and follows the proposed nonlinear learning procedure. We adopted an intensity-induced phase nonlinearity: $y=x\cdot\exp^{1j\left| x \right|^{2}}$, and trained a 4-layer nonlinear neural network on MNIST dataset. Compared with the linear neural network, which achieved a classification accuracy of 90.4%, the classification accuracy increased to 93.0% with nonlinear neural network, which proves the effectiveness of the proposed nonlinear FFM learning framework. We have also trained four-layer neural networks on more challenging CIFAR and ImageNet datasets for four-category classification, the results are shown in Fig. 2g. The experiments yielded accuracies of 60.0% and 59.5%, respectively, closely aligning with in-silico trained accuracies of 63.5% and 61.0%. Numerical evaluation validated that further increasing the number of layers from 4 to 8 would enhance the classification accuracies to 67.3% and 64.2%, respectively.

# Supplementary Note 12. Symmetrical neural networks

Here we numerically prove that the weight matrix supported by symmetrical propagation have similar performance to the multi-layer fully connected neural network as long as they have similar number of trainable weights.

We first illustrate that even though the propagation media is symmetrical, the weight matrix is not. Denote the propagation matrix and the modulation matrix as $W$ and $M$, where $W=W^{T}$ and $M$ is a diagonal matrix. So, the transpose of the weight matrix is $\left( WM \right)^{T}=M^{T}W^{T}=MW$. For non-trivial $M$ that is not identity matrix, $MW\neq WM$, so the weight matrix $WM$ is not necessarily symmetrical.

We then compared the performances of the neural network with and without the symmetrical propagation on Iris dataset and MNIST dataset. The results are shown in Fig. S20. We changed each 2×2 connection in the PIC neural network depicted in Fig. S19, from symmetrical to fully connected (FC). The number of parameters was increased from 64 to 128. It is observed that the integrated network had the same accuracy of 96.67%. We further evaluate the symmetry on more complex MNIST classification task. Just like the evaluation on Iris dataset, the input dimension of the MNIST image was down-sampled to 16, and the output dimension was 8 (for 8-class handwritten image classification, 8000 data was used for training and 800 data was used for testing). We employ the five-layer fully connected (FC) networks with 16, 16, 16, 8, 8, output neurons for comparison. The total number of parameters was 896. In FFM, we compare two network architecture, the first is FFM-16, with the same input neuron number while having only 64 parameters. The first is FFM-256, where the input neuron numbers were increased to 256, 256, 256, 64, and 64 to keep the number of parameters the same as that of the FC. For each layer of the FFM, the input was amplitude modulated with parameters and connected to the next layer with a random symmetric propagation. Moreover, the input and output dimension of FFM-16 network was the same to FC network. In FFM-256, to maintain consistency between the input of FFM-256 and FC network, the input of the FFM-256 network $I_{\mathrm{FFM}}$ was 16 times repeated from the input of the FC network $I_{\mathrm{FC}}$, which can be written as $I_{FFM}=M_{I}I_{FC}, M_{I}=\left[ \mathbf{e}_{\mathbf{1}}\boldsymbol{\otimes1,}\mathbf{e}_{\mathbf{2}}\boldsymbol{\otimes1,...,}\mathbf{e}_{\mathbf{16}}\boldsymbol{\otimes1} \right]^{T})$, where $\mathbf{e}_{\mathbf{i}}$ is the 16-d singleton column vector, $\mathbf{1}$ is the 16-d all-ones row vector and $\boldsymbol{\otimes}$ represents the outer product. Through five repetitions of the entire training procedure, the average results are depicted in Fig. S20. Notably, it is observed that the FFM-256 and FC network exhibit similar test accuracies, specifically 87.5% and 87.0% respectively and the FFM-16 network achieved 81.8%. Subsequently, upon incorporating Rectified Linear Unit (ReLU) nonlinearity between layers, the classification accuracy of FFM-256, FFM-16 and FC increased to 89.7%, 83.5% and 89.1% respectively. FFM networks demonstrated comparable performance to FC networks with similar number of parameters. The symmetry of the propagation thus does not impose limitations on the computing capability of neural networks trained with FFM learning. Enhancing the classification accuracy can be achievable through the utilization of deeper and wider layers. As demonstrated in Fig. S20 (c), by expanding the layers to 8, with output neuron configurations of 512, 512, 128, 128, 32, 32, 8, and 8, the accuracy escalates to 96.13%.

# Supplementary Note 13. Onsite calculation of loss and gradient

As illustrated in Supplementary Note 11, the majority of operations for training have been experimentally implemented on site. Here we explain how the rest of operations can be performed on site as well. As shown in Fig. S6. The loss calculation and gradient compute can both be implemented with onsite optics or electronics.

The loss calculation involves the operation of minus, which can be achieved through a $\pi$ phase shift (equivalent to the value of -1 in the complex representation of light), and interference, which calculates the difference between the output $y$ and the target $T$, $e=y-T$.

From Eq. S13, the gradients of the parameters $g\propto\left( Re\left\{ {1jE}_{data}E_{error} \right\}-Im\left\{ {1jE}_{data}E_{error} \right\} \right)$. So that the gradient compute involves calculating the real and imaginary parts of the field $E_{data}E_{error}$. To compute $Re\{{1jE}_{data}E_{error}\}$, the intensity of three fields are detected: ${A=\left| E_{data}^{*}+1jE_{error} \right|}^{2}$, $B=\left| E_{data} \right|^{2}$, $C=\left| E_{error} \right|^{2}$, and the gradient sums to $Re\left\{ {1jE}_{data}E_{error} \right\}=(A-B-C)/2$, where the phase conjugation can be realized by a phase conjugation mirror ^18^, and the difference operations can be realized with balanced photodetection. Similarly, the intensity ${A'=\left| E_{data}^{*}+E_{error} \right|}^{2}$ can be measured, then we have $Im\left\{ {1jE}_{data}E_{error} \right\}=(A^{'}-B-C)/2$. The detected gradient signals will be further accumulated through integral circuits ^19^ to drive the modulator and tune the refractive index. The gradient may also be used for direct update of the refractive index of non-volatile optical materials^20^.

# Supplementary Tables.

Table S1. Summary of the experimental FFM learning efficiency and performance.

| Tasks  Methods | Deep neural network | Imaging through scattering media | NLOS imaging  (Amplitude-target) | NLOS imaging /processing  (Phase-target) | Integrated photonic  neural network | |
| --- | --- | --- | --- | --- | --- | --- |
| FFM learning with  commercial SLM | < 2 ms/Iter.  3000 Iter. to converge  (92.5% Acc. F-MNIST) | < 2 ms/Iter.  25 Iter. to converge  (81.2µm FWHM) | < 2ms/Iter.  100 Iter. to converge  (1.0 SSIM) | < 2 ms/Iter.  20 Iter. to converge  (0.81 SSIM) | | N/A |
|  |  |  |  | < 2 ms/Iter  6000 Iters to converge  (1.0 photon/pixel @ 94.5% Acc. MNIST) | |  |
| FFM learning with  MEMS VOA | N/A | | | | | 64 ms/Iter.  600 iters to converge (94.2% Acc. IRIS) |
| FFM learning with ultrafast SLM  (Projected) | <2.0 ns/Iter | | | | | N/A |
| FFM learning with  ultrafast MZM | N/A | | | | | 64 ns/Iter |

Table S2. Workload of FFM error propagation with different AI architectures.

| Methods | Batch Size  (BS) | Number of layers  (N) | Workload  (BS/(BS+N-1)) | Source |
| --- | --- | --- | --- | --- |
| 8-layer ONN | 10 | 8 | 58.8% | This work |
| Residual Network | 256 | 56 | 82.3% | Ref^4^ |
| Generative pre-trained transformer-3  (GPT-3) | 3.2 million | 96 | 100% | Ref^16^ |

# Supplementary Figures S1-S18


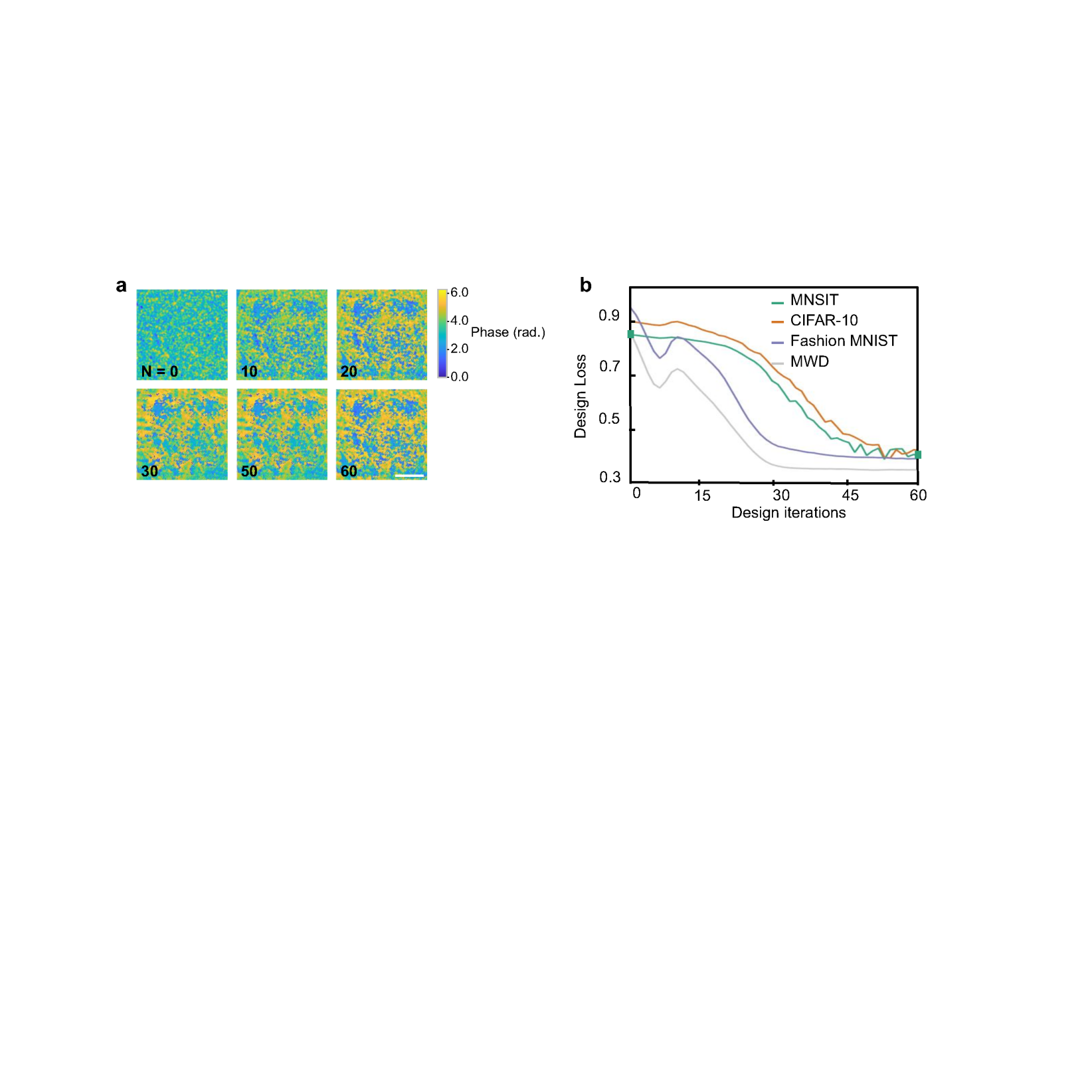


**Fig. S1.** **a** Parameters evolution of the FFM learning. We train one reconfigurable phase mask as design space for the classification task on the MNIST dataset. It can be observed that as the FFM design progresses, the design space gradually converges. Scalebar: 1 mm. **b** FFM learning on different benchmarking datasets.


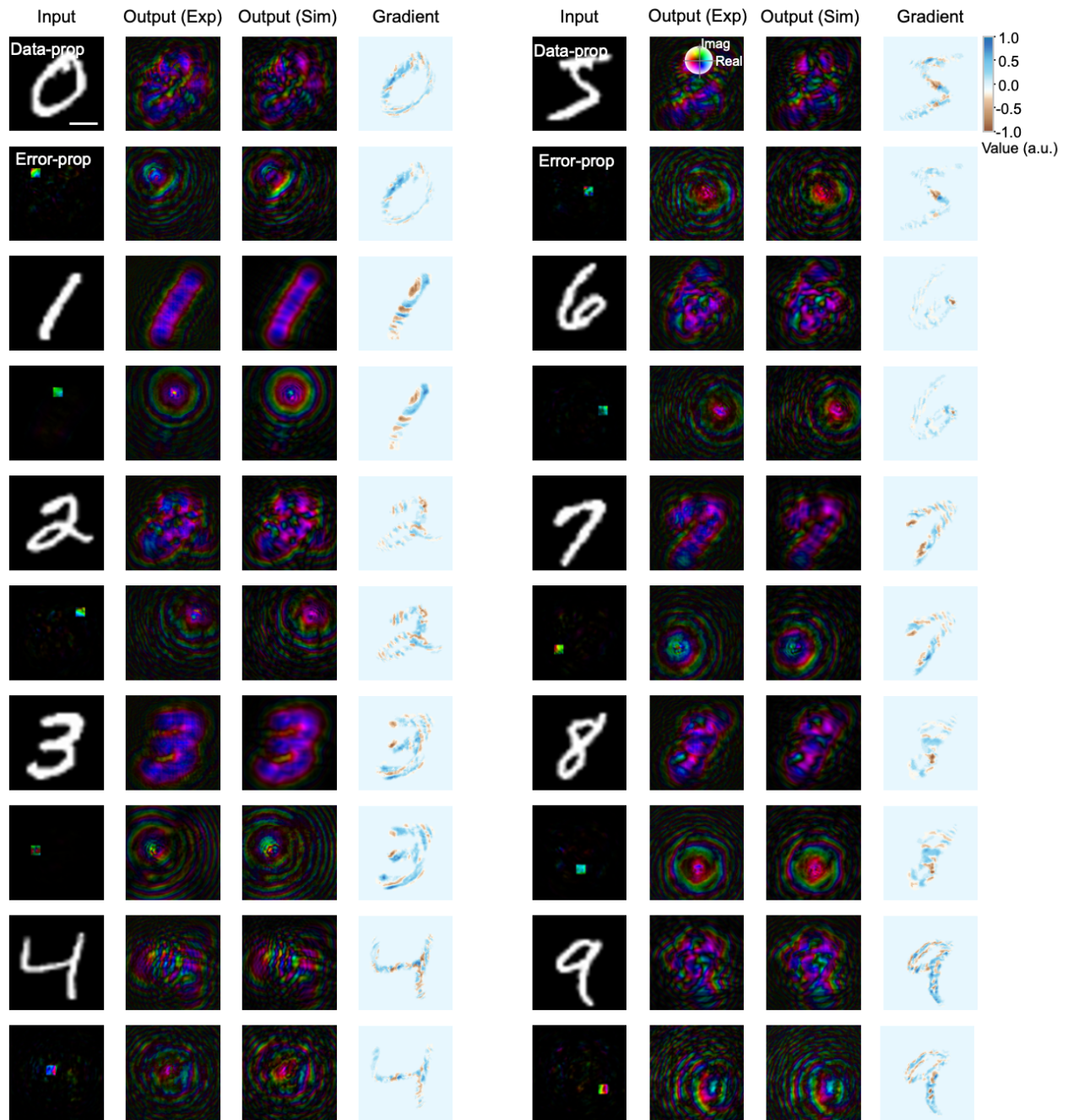


**Fig. S2.** Experimental measurements compared with analytical simulation (supplementary to Fig. 2 of main text). Here are samples for each category in MNIST dataset. For each number we compare the complex field of data propagation and error propagation and the gradient which is calculated by two forward propagation results. The experimental measurements are very close to the analytical simulation results which demonstrates the high fidelity of the FFM system.


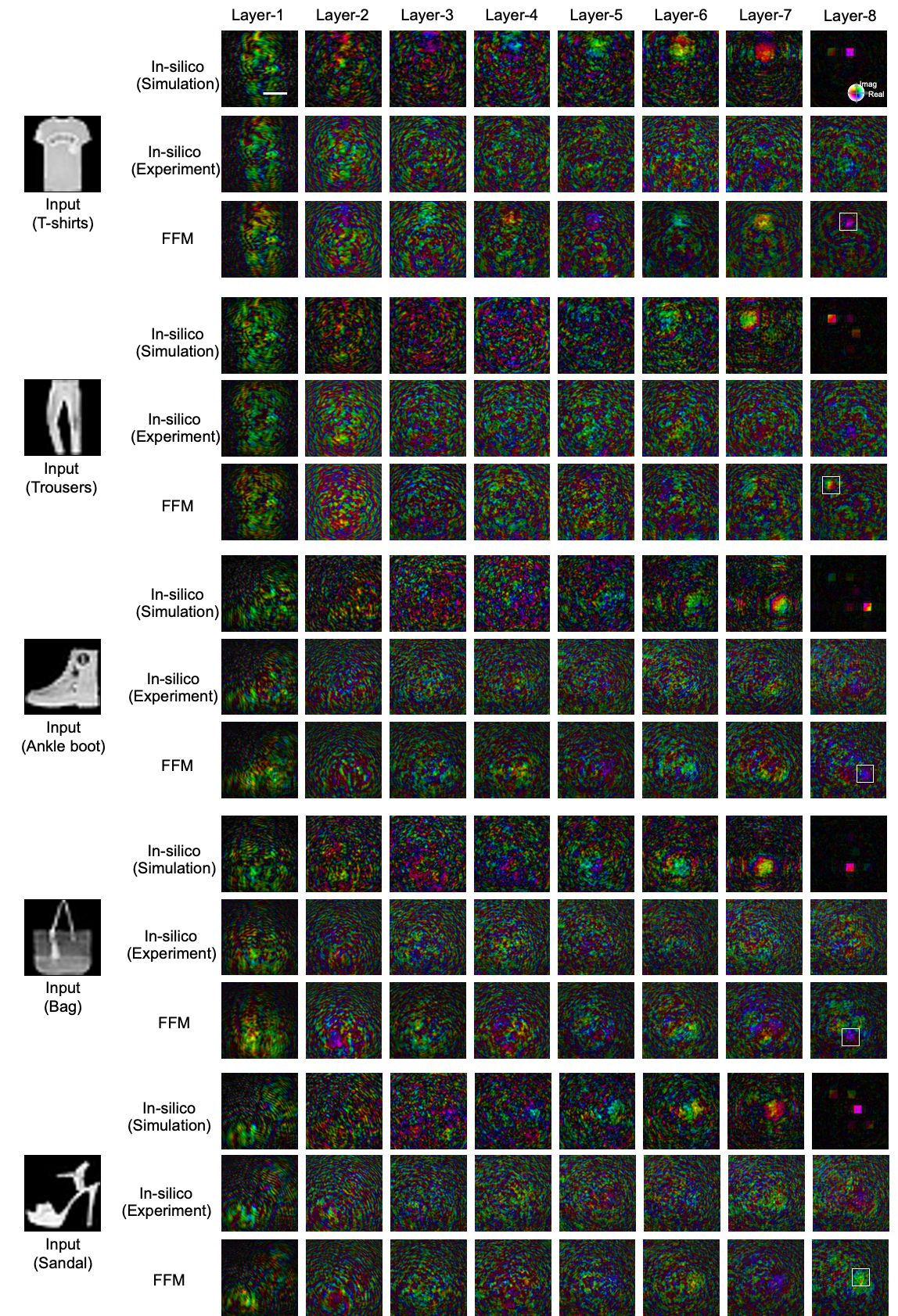


**Fig. S3.** Per-layer outputs of an 8-layer ONN designed with FFM learning compared with in-silico optimization and simulation ground-truth (supplementary to Fig. 2 of main text). It can be observed that the FFM learning shows a gradual convergence towards the target regions in each layer's output, which is similar to the ground truth results. This indicates that in the 8-layer ONN network, FFM is capable of optimizing each layer to contribute to the classification task and achieve the desired outcome. On the other hand, in the in-silico optimization results, the outputs of the first two layers are relatively close to the ground truth. However, as the number of layers increases, the errors accumulate, leading to significant deviations in the subsequent outputs and the inability to achieve classification. Here are samples for 5 categories in Fashion-MNIST dataset.


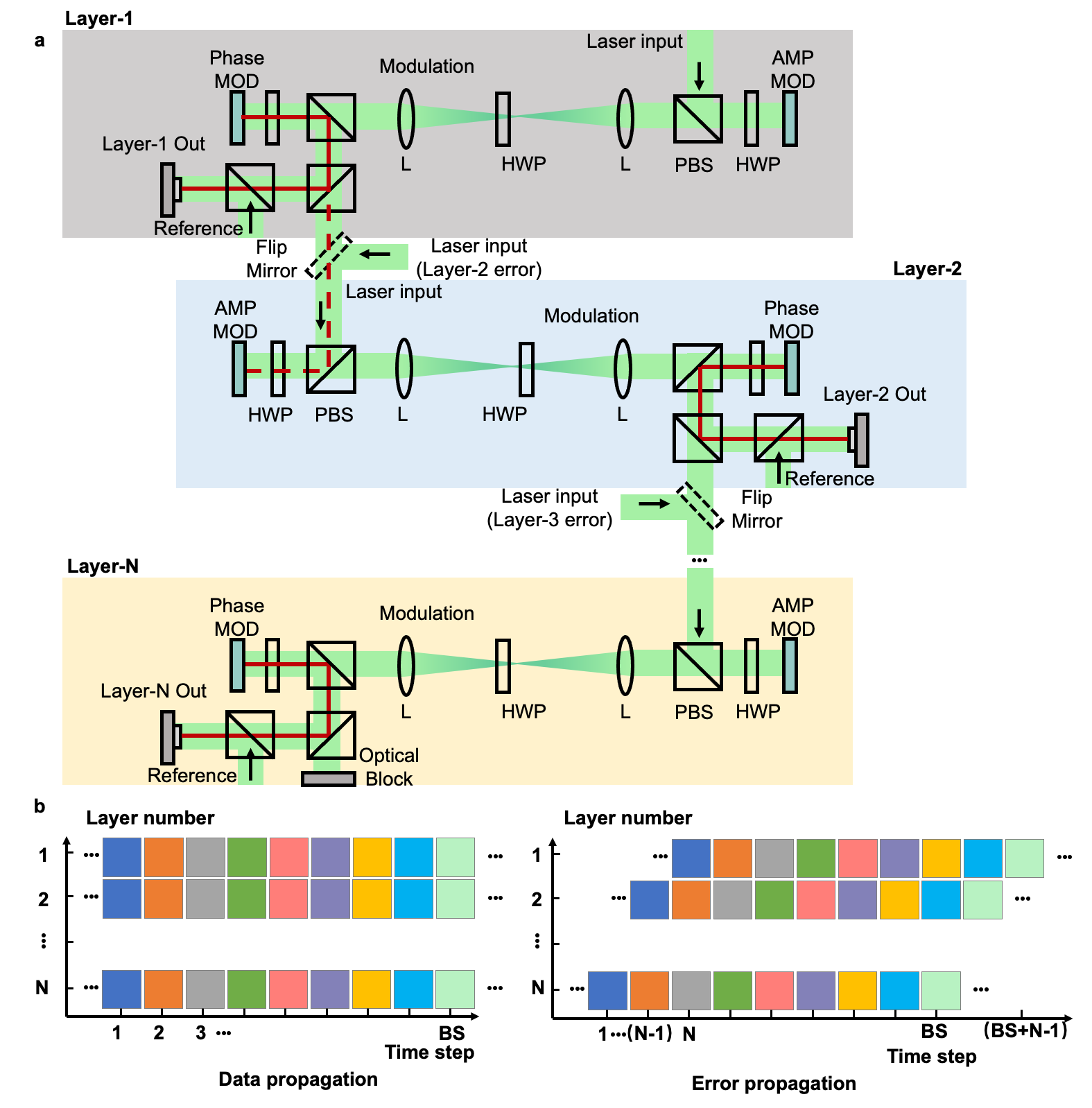


**Fig. S4** The proposed multilayer training system (a) and the associated learning streamline (b).


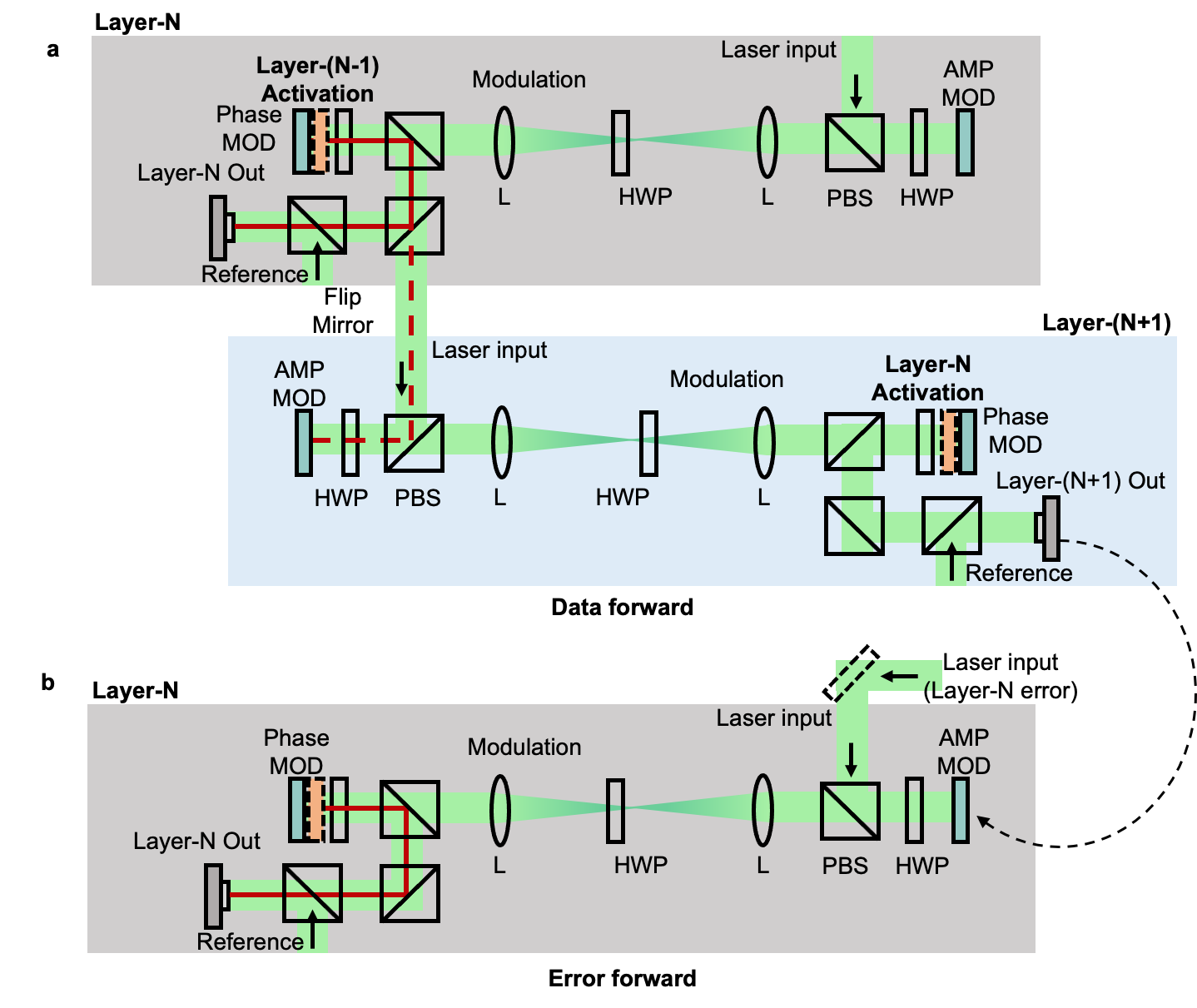


**Fig. S5.** FFM learning with nonlinear activation. **a,** The data propagation. **b,** The error propagation.


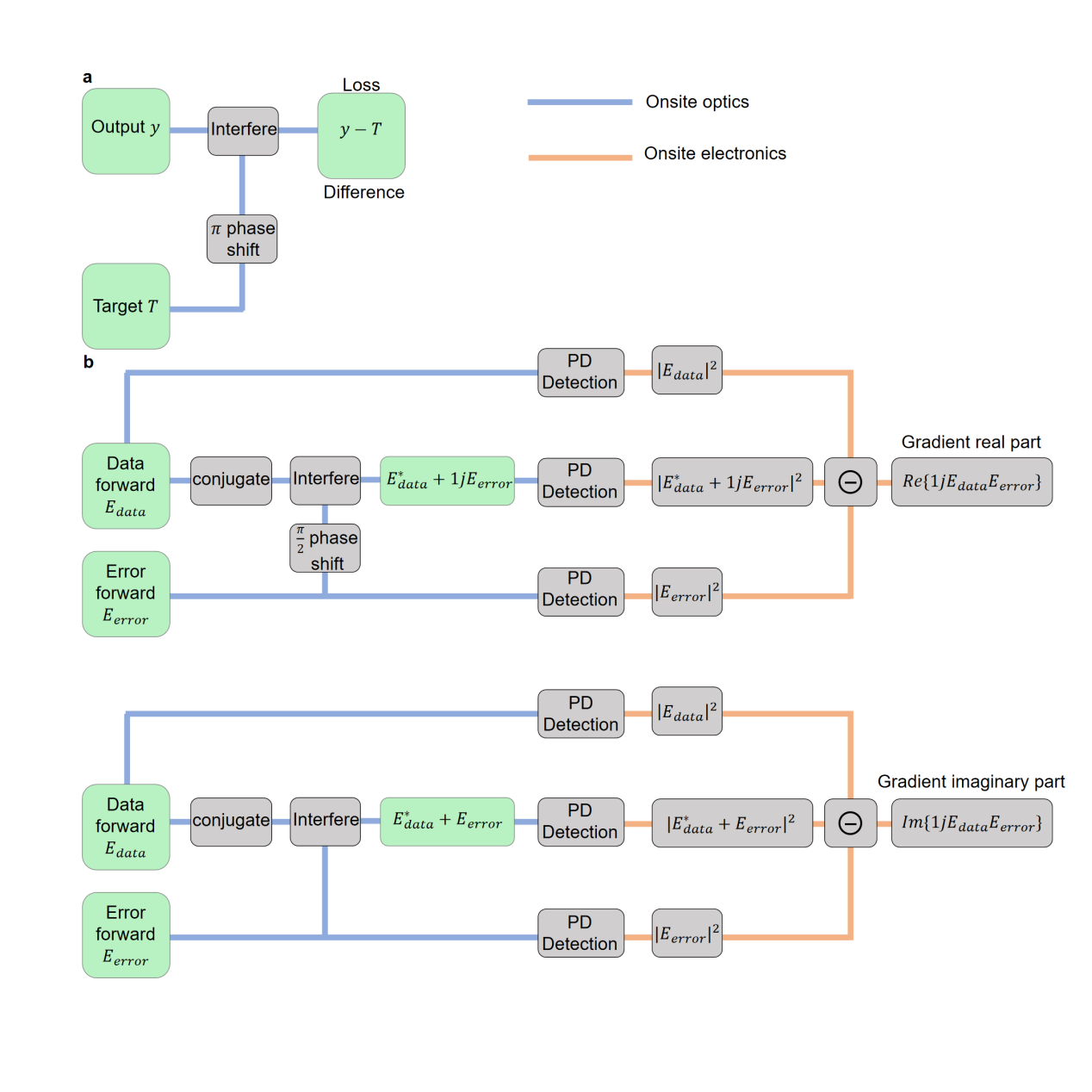


**Fig. S6.** Onsite optics and electronics for calculating the loss (a) and parameter gradient (b).


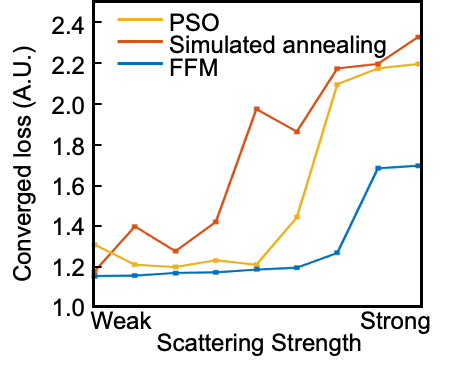


**Fig. S7.** Focus optimization with different levels of type-I scattering media. By applying a Gaussian kernel with varying standard deviations (0.9, 0.8, 0.7, 0.6, 0.5, 0.4, 0.3, 0.2, 0.1-pixel) to a completely random phase mask, we generate phases corresponding to different levels of scattering strength. We compare the FFM with two in-situ optimization algorithms: particle swarm optimization (PSO) and simulated annealing (SA). It can be observed that FFM achieves the lowest design loss than optimization algorithm in different levels of scattering. As the PSO achieves lower loss, we use PSO for comparison in the main text.


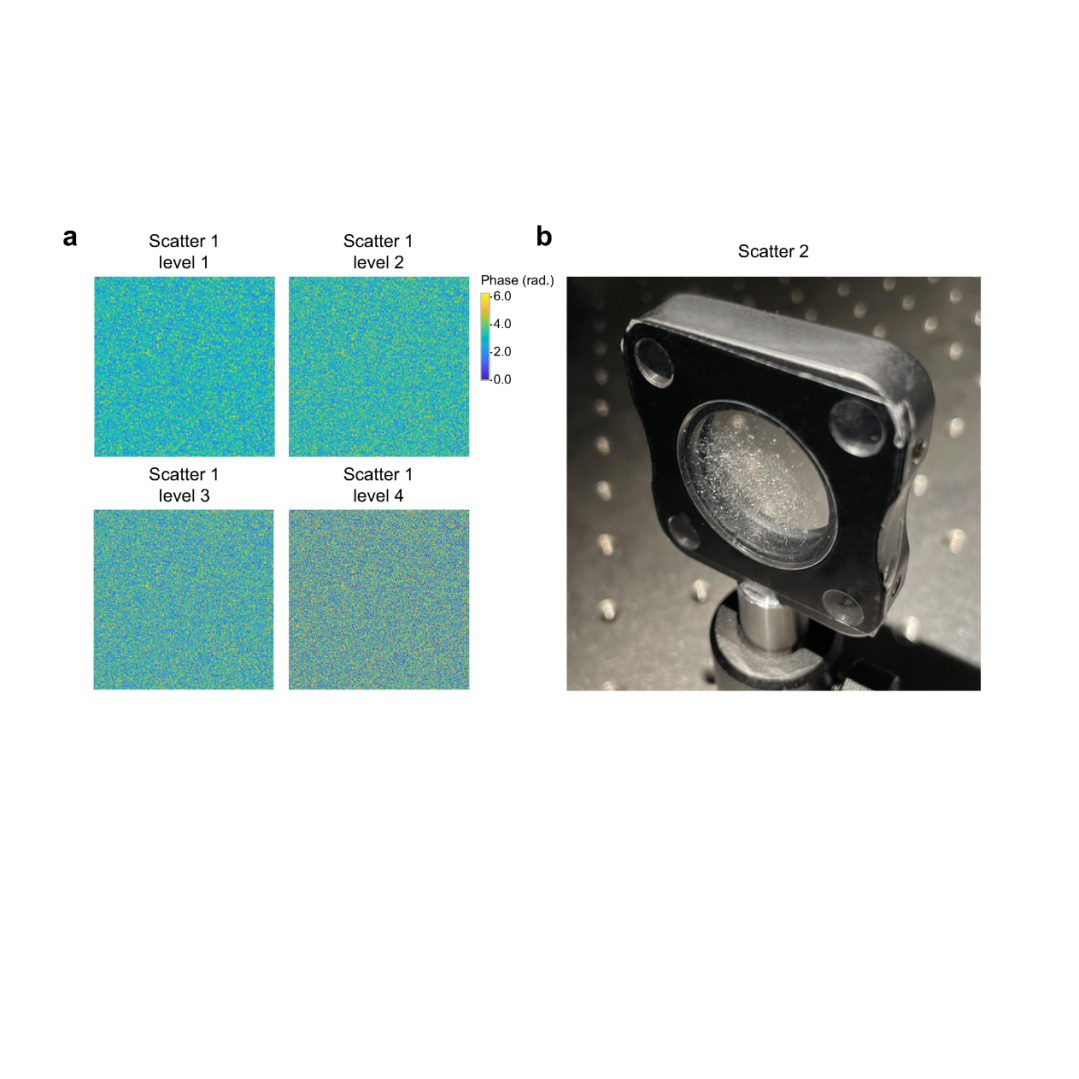


**Fig. S8.** Scattering realization. Scattering mask in scatter-1, from level-1 to level-4 (**a**) and used scotch tape for scatter-2 (**b**).


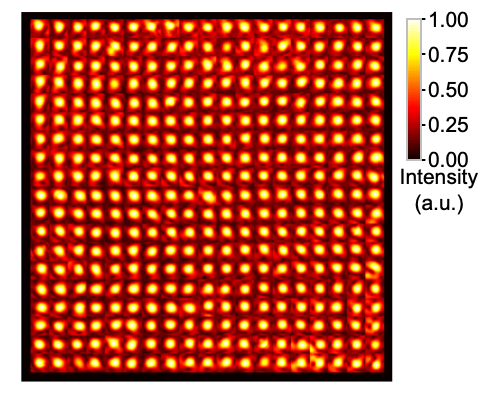


**Fig. S9.** 20×20 optimized foci array over a 3.2 mm × 3.2 mm area.


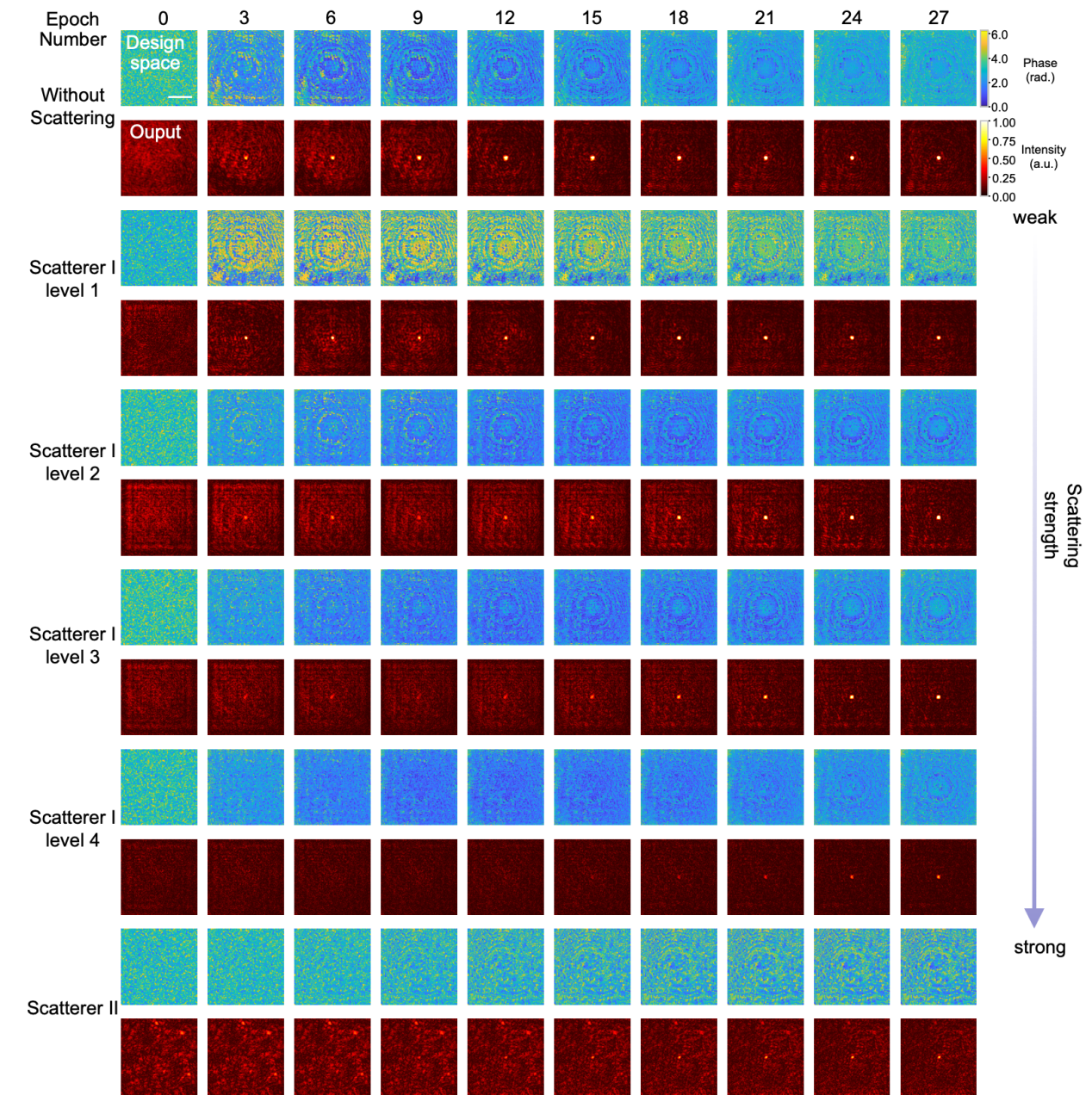


**Fig. S10.** Focus optimization process with different levels of scattering and different types of scatterers with FFM learning (Supplementary to contents in Fig. 3). The changes of design space and output with convergence are shown for each scattering. Scatterer-I is realized by random phase masks on a SLM while Scatterer-II is realized by a scotch tape with random pattern on it. For scatter 1 there are four levels of scattering with increasingly stronger scattering. The scattering level of scatterer-I is determined by the variation in phase of a random phase mask. By applying a Gaussian kernel with different standard deviations (0.9, 0.7, 0.5, 0.1) to a completely random phase mask with per-pixel phase uniformly sampled between 0 and 2$\pi$, we obtain random phases with four corresponding levels of scattering with increasing scattering strength. The scatterers are visualized in Fig. S8.


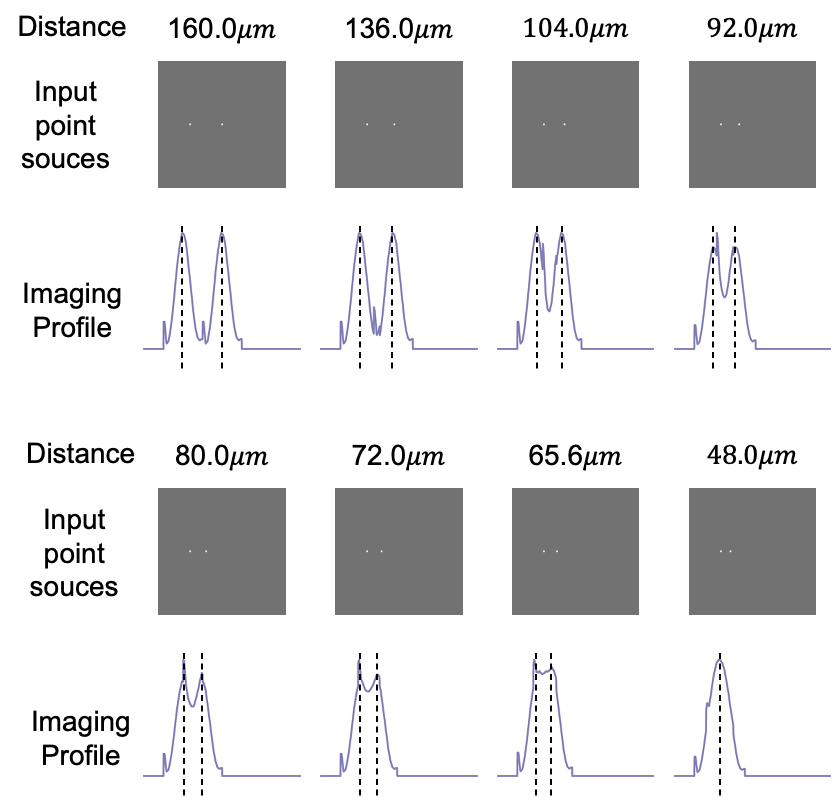


**Fig. S11.** Resolution limit of imaging with FFM learning. Given input consisting of only two point sources, we convolve the input image with the optimized focal point to obtain the imaging result. The output here represents the central profile of the imaging result. It can be observed that as the distance between the two point sources gradually decreases, the two points in the imaging result become increasingly difficult to distinguish. Especially at a distance of 65.6 $\mu m$, the two points are almost indistinguishable. From this we can deduce that the FFM learning provides imaging of the focal points with a resolution of approximately 65.6 $\mu m$.

**
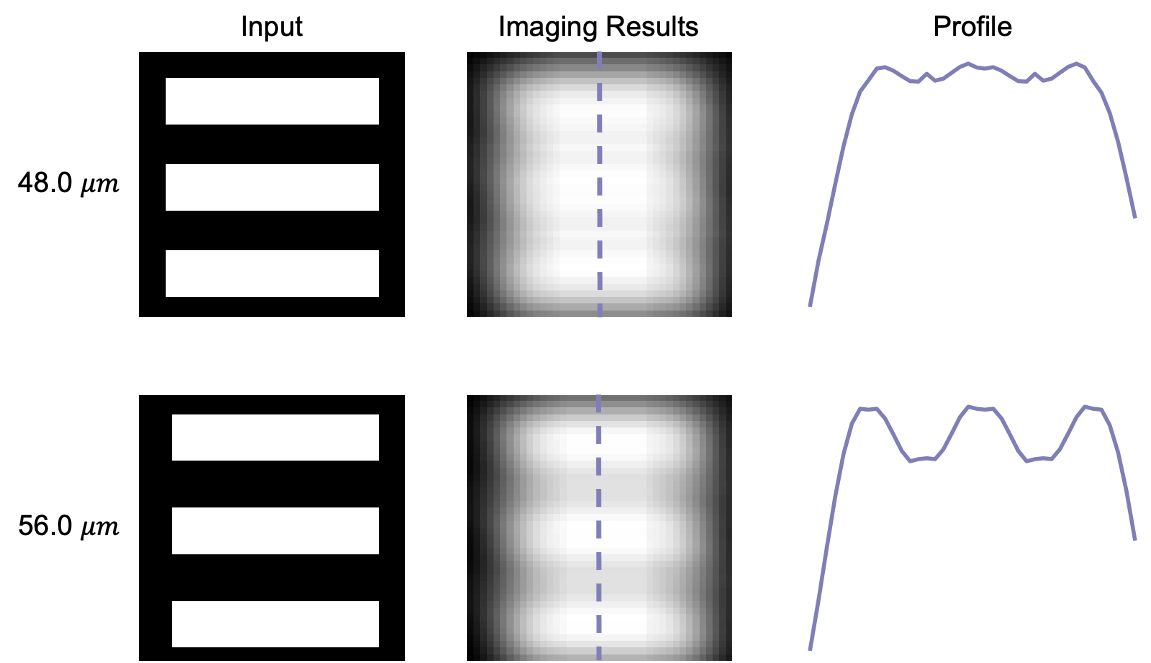
**

**Fig. S12.** Visualization of resolution limit of imaging on testing chart. Here, we compared the imaging results of inputs with line width of 56.0 µm, and line spacings of 48.0 $\mu m$ and 56.0 $\mu m$, respectively. It is evident that when the separation is 56.0 $\mu m$, the lines in the imaging result are distinguishable, whereas at 48.0$\mu m$, the lines become completely indistinguishable.


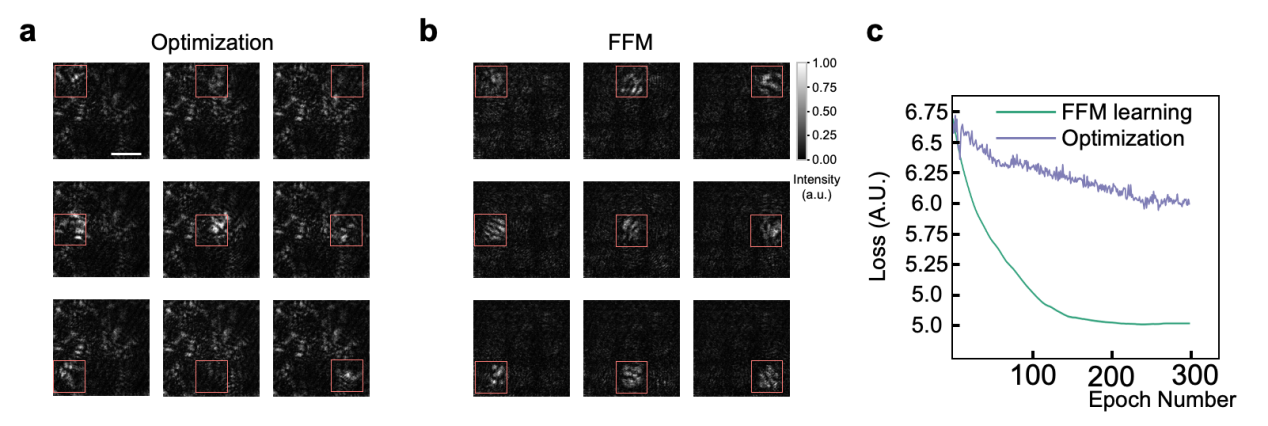


**Fig. S13.** Training results of the imaging region using the optimization algorithm (**a**) and FFM learning (**b**), with convergence trends displayed in **c**. It can be observed that the FFM learning exhibits higher signal-to-background ratio compared to the optimization algorithm.


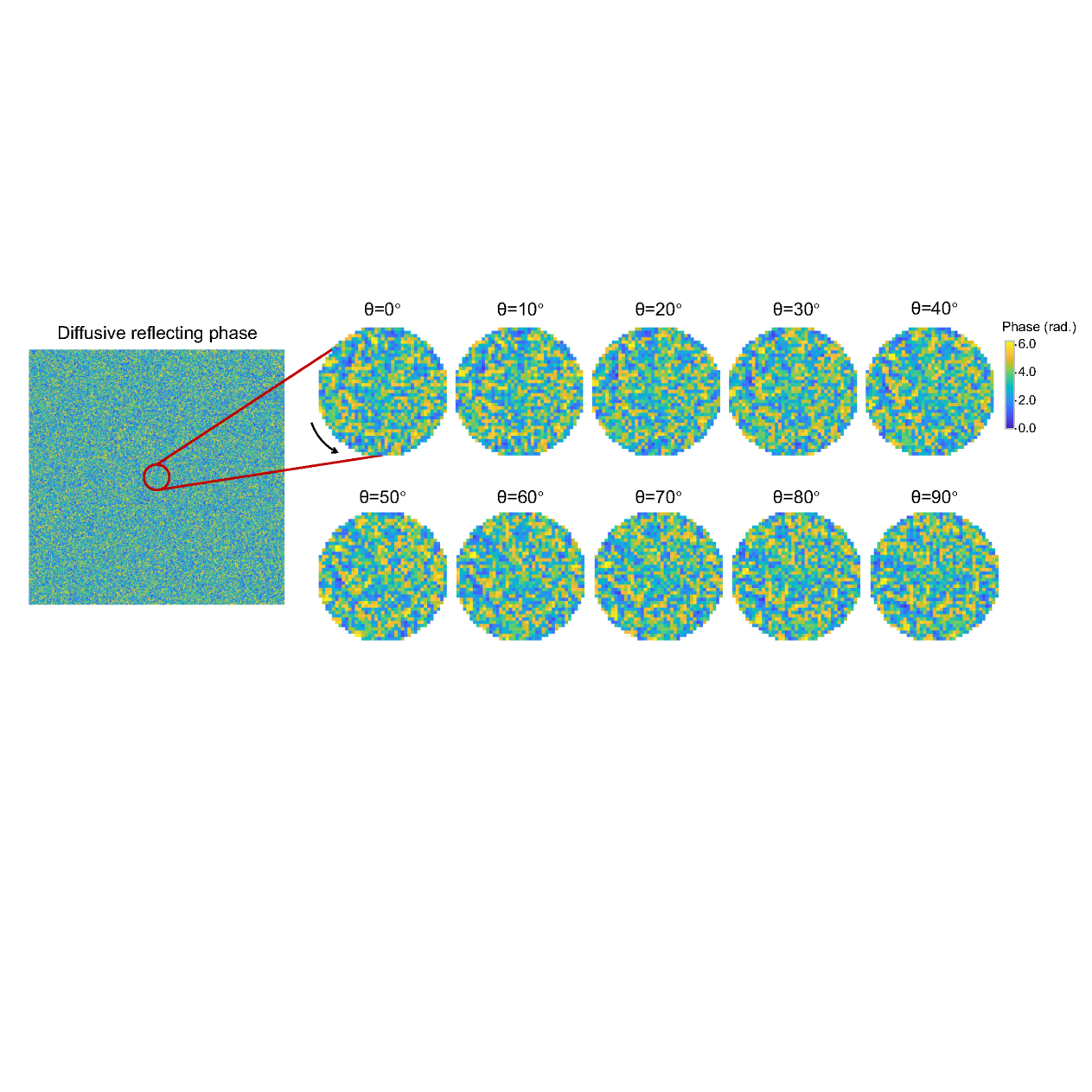


**Fig. S14.** Visualizations of dynamic scattering media. In the dynamic NLOS classification, the diffusive reflecting phase has two rotating speeds, the slow speed was 1° per iteration and the faster speed was 2.5° per iteration.


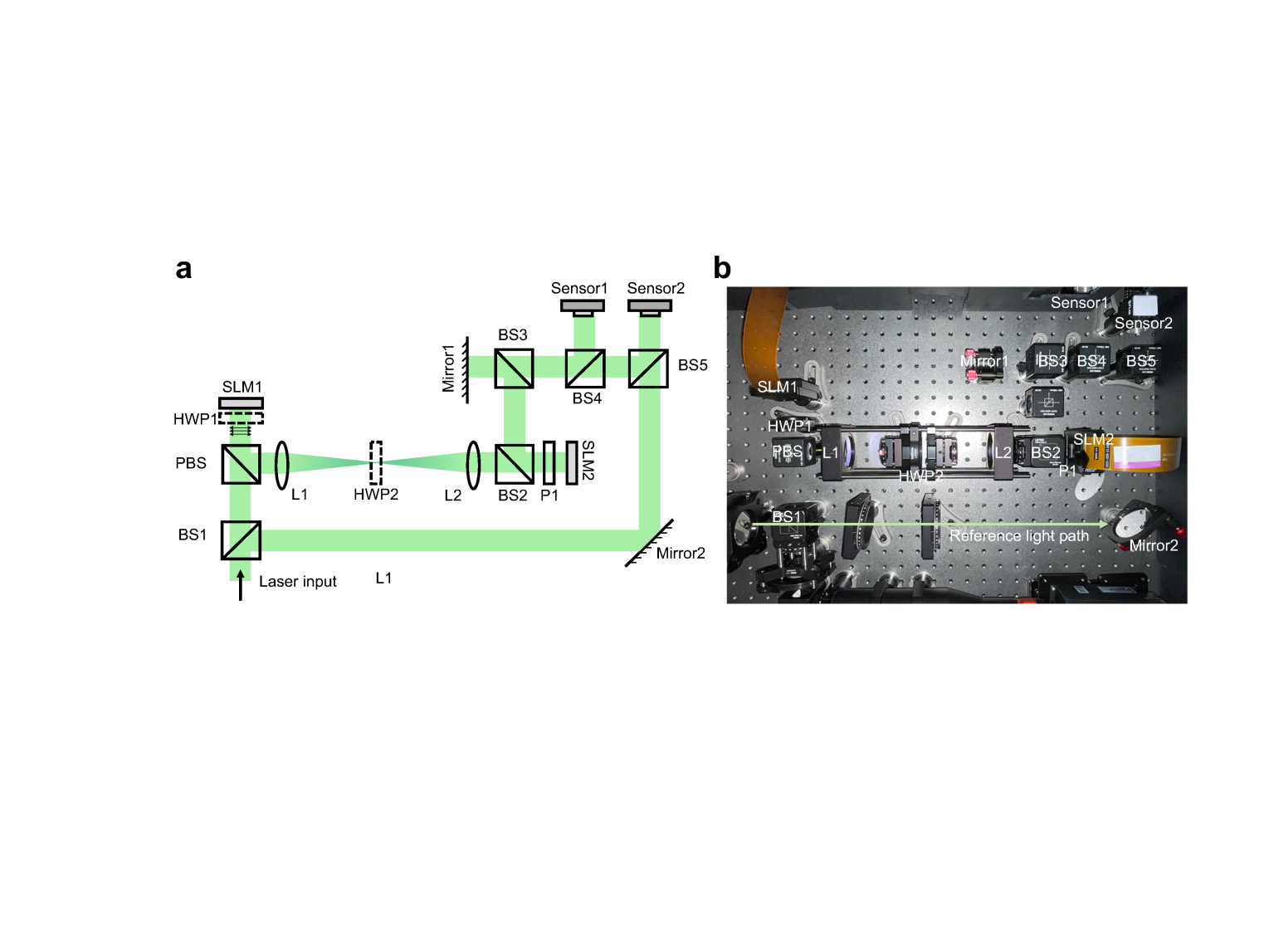


**Fig. S15.** Schematic (**a**) and the picture (**b**) of the experimental free-space optical system. L1, L2, relay lenses; SLM1, SLM2, spatial light modulators; P1, polarizer; HWP1, HWP2, half wave plates; BS1, BS2, BS3, BS4, BS5, beam splitter; PBS, polarized beam splitter. The laser input is optically modulated by the SLMs for multi-layer photonic computing. SLM1 is used to for input loading and SLM2 is used as the designed space. A reference light path is used for complex field measurement.

**Fig. S16.** Experimental system setup of the non-line-of-sight scene. **a,** For amplitude imaging, the system was set up with diffusive reflector and hidden target. **b,** The optical image of the system in (a). The modulated input light entered the NLOS region after being reflected with the diffusive reflector, which composed of a processed scotch tape (Fig. S8(b)) and a reflector. The input light incident on the chromium target and propagated back to the line-of-sight regime. **c,** For phase imaging and all-optical processing, in order to accommodate with the large training data number, the diffusive reflector and the targets were combined and projected onto the SLM. The diffusive reflector was emulated with random phase patterns uniformly drawn from uniform distribution **[**0, 2$\pi$**]** independently per pixel, and the 8-bit grayscale objects were linearly mapped to 0-$\pi$.


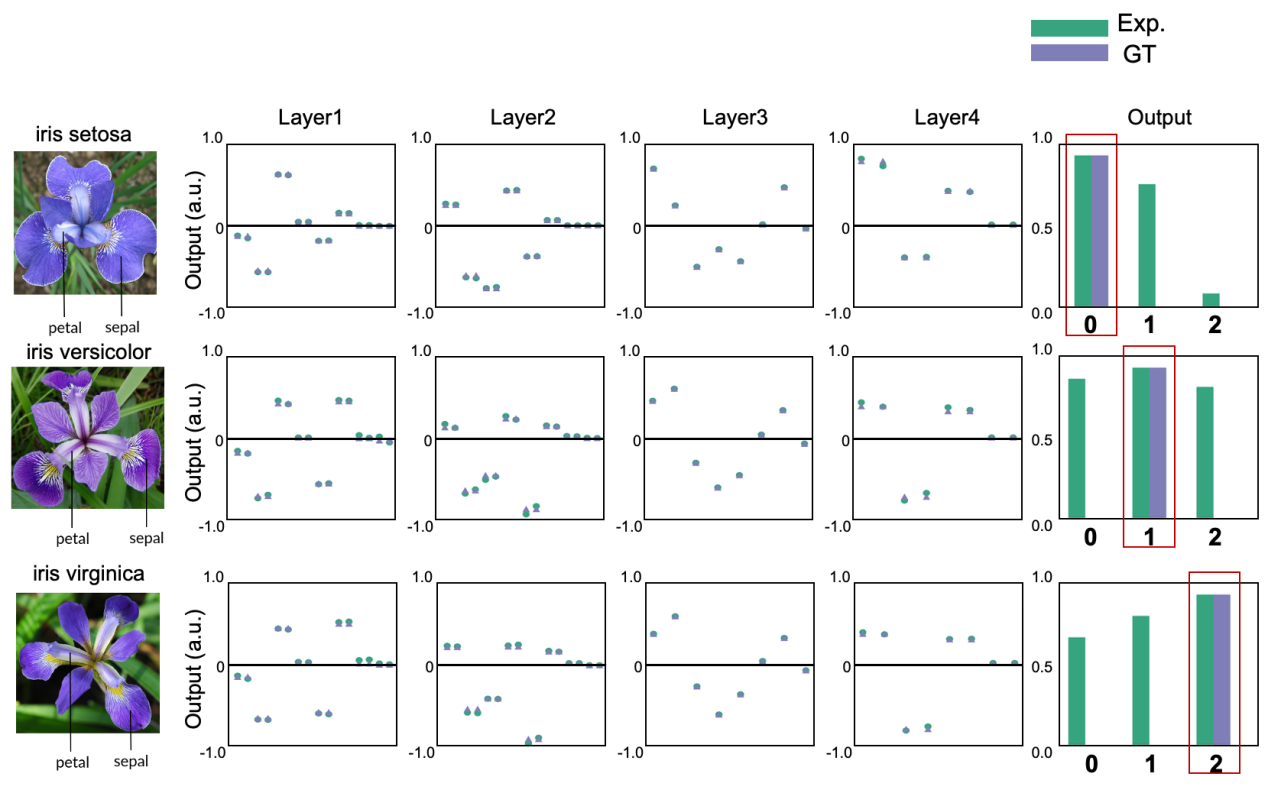


**Fig. S17.** Visualization of input data and intermediate outputs of Iris dataset classification (supplementary to Fig. 5 in the main text). The input data is a 4-channel tensor which indicates 4 characteristics of the Iris flower and resampled to 16-D for PIC network input. For each layer, the outputs of the experimental and simulation results are shown in scatterplot and the experimental results have minimal distinction from the ground-truth.

**
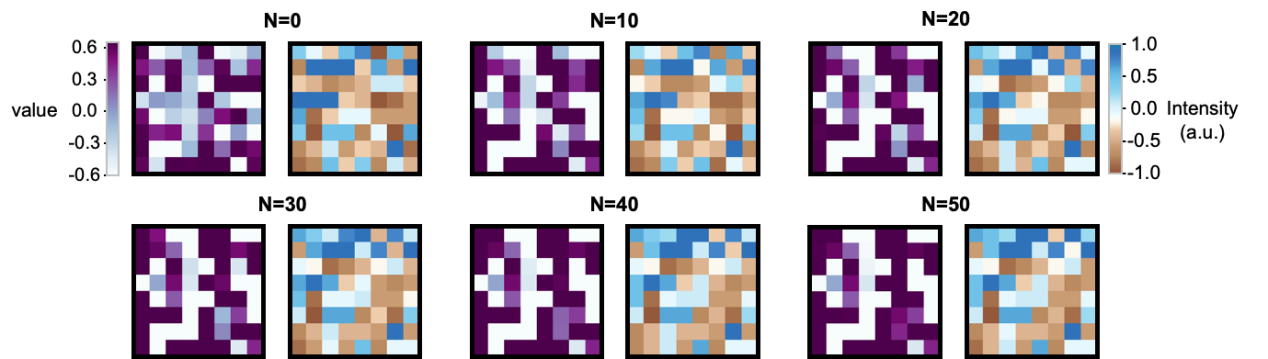
**

**Fig. S18.** Parameters evolution of the FFM learning and its corresponding gradient on PIC neural network (supplementary to Fig. 5 in the main text). We utilize 64 parameters of 5 layers (16,16,16,8,8) as design space for training a classification task on the Iris dataset. Here we flatten the parameters and gradient into an 8×8 matrix. It can be observed that as the FFM learning progresses, the design space gradually converges.


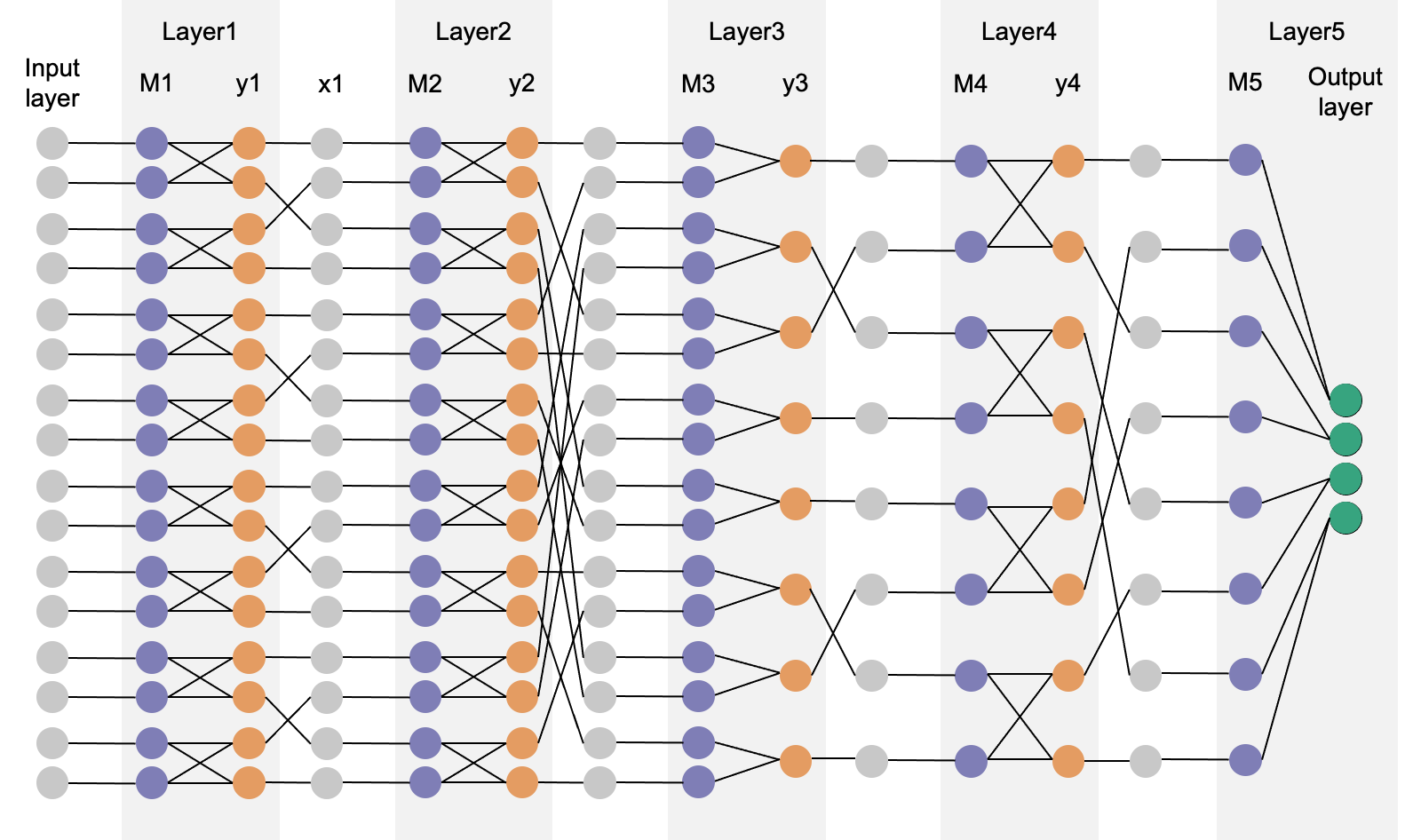


**Fig. S19. PIC neural network architecture.** The whole PIC neural network contains five layers. Measurements are shuffled before being fed into subsequent layer. The 5-layer neural network is 32 times the scale of the symmetry core.


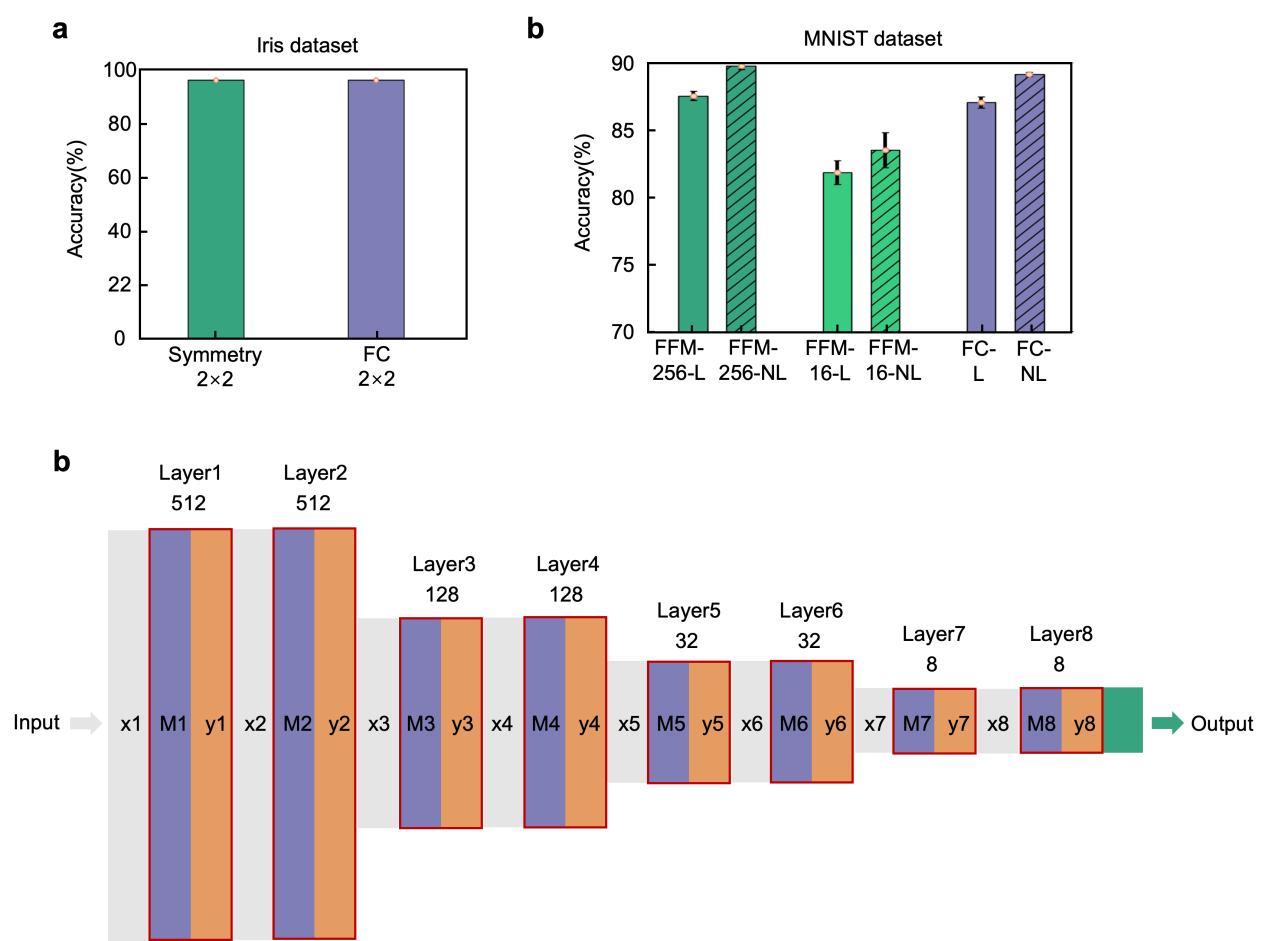


**Fig. S20.** Symmetry evaluation on Iris dataset (a) and MNIST dataset (b). Error-bar length delineates two times the standard deviation. (c) Scaled 8-layer FFM network with 512, 512, 128, 128, 32, 32, 8, and 8 output neurons, the accuracy can be increased to 96.13%.


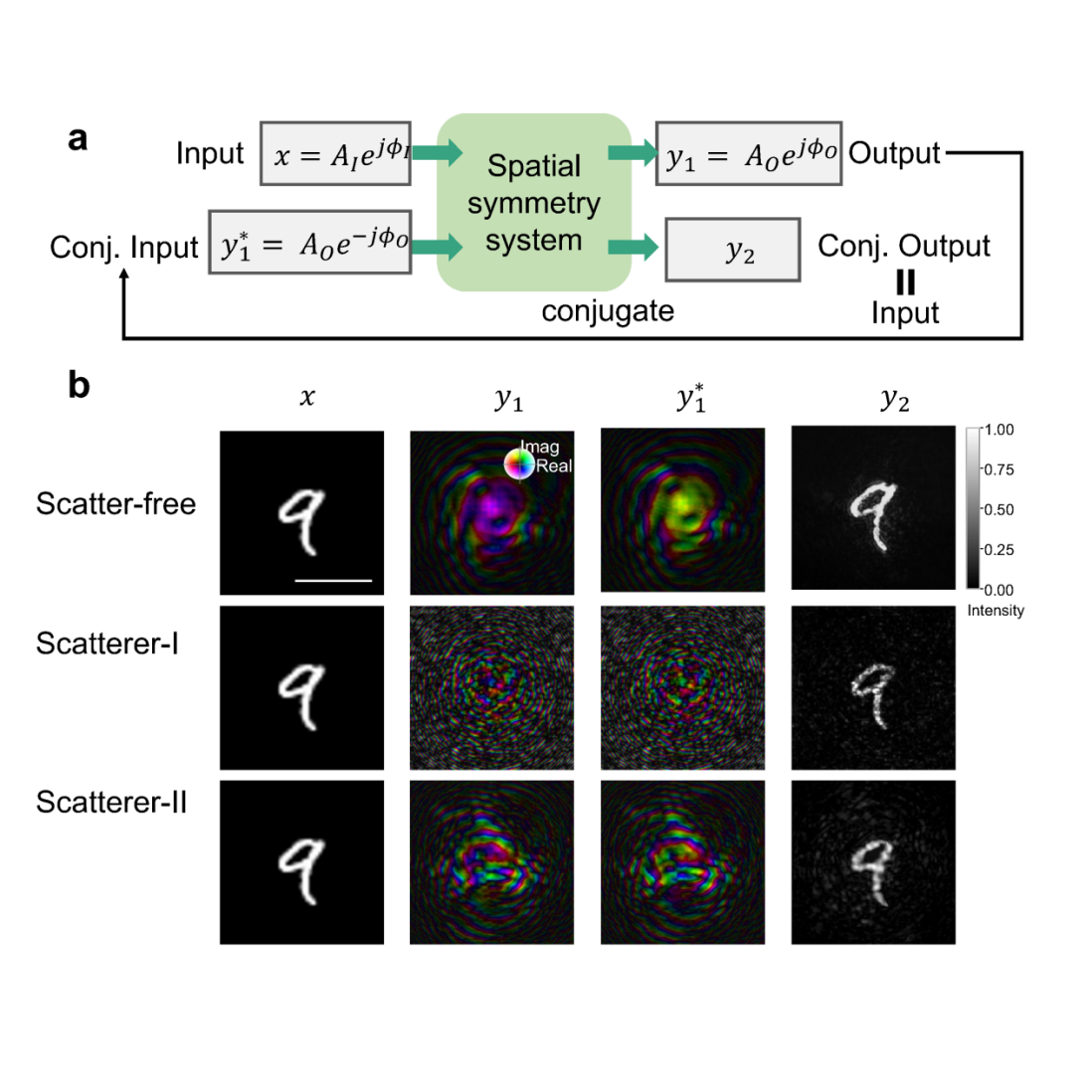


**Fig. S21.** Validation of spatial symmetric reciprocity. **a** In symmetrical system, the conjugation and forward propagation of the outputs would approximately reproduce the input, which can be used for determined the spatial symmetry. **b** The measured conjugated outputs in three types of propagation media. The letter "9" are reimaged after the second propagation. Scalebar: 1 mm.

**
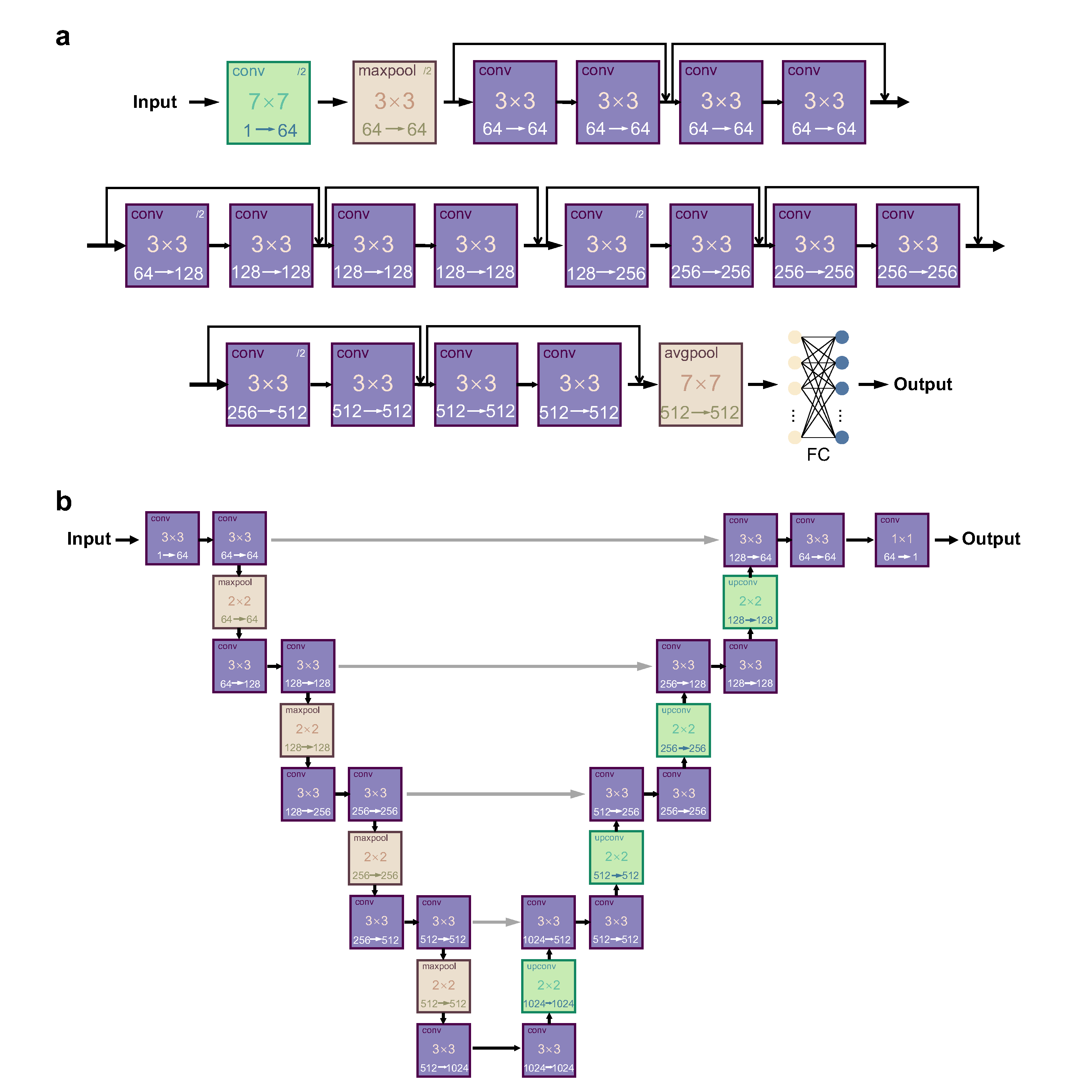
Fig. S22.** Network architecture for comparison. **a** Residual network. Each square block in the figure is one convolution or pooling operation. The numbers in the center of the square block represent the kernel size and the numbers with arrow at the bottom of the square block means the channel transform of this operation. If there is '/2' is in the upper right corner of the square block, the stride of the operation is 2 and the output size will be cut in half. The arrow above square blocks represent the jump across layers used for residual calculation. **b** U-Net. Square blocks in the figure are the same as those in residual network. The gray arrows mean copy and crop the output from the down-sample part to a corresponding up-sample part.

# Supplementary video descriptions

Video S1. A video displaying the process of imaging through scattering media by scanning the image plane with local focal point and comparing the imaging results of FFM learning with optimization method.

Video S2. A video displaying the process of imaging in NLOS scenes. It shows the learning process with square grid regions and testing process of imaging dynamic chromium letter targets ("T", "H", and "U").

# References

1. Goodman, J. W. *Introduction to Fourier optics*. Roberts and Company publishers (2005).

2. Zheludev, N. I. & Kivshar, Y. S. From metamaterials to metadevices. *Nature materials* **11**, 917-924 (2012).

3. Joannopoulos, J. D., Johnson, S. G., Winn, J. N. & Meade, R. D. Molding the flow of light. *Princet. Univ. Press. Princeton, NJ [ua]*, (2008).

4. He, K., Zhang, X., Ren, S. & Sun, J. Deep residual learning for image recognition. In: *Proceedings of the IEEE conference on computer vision and pattern recognition* (2016).

5. Ronneberger, O., Fischer, P. & Brox, T. U-net: Convolutional networks for biomedical image segmentation. In: *International Conference on Medical image computing and computer-assisted intervention* (2015).

6. Kirkpatrick, S., Gelatt Jr, C. D. & Vecchi, M. P. Optimization by simulated annealing. *science* **220**, 671-680 (1983).

7. Kennedy, J. & Eberhart, R. Particle swarm optimization. In: *Proceedings of ICNN'95-international conference on neural networks* (1995).

8. Shirmanesh, G. K., Sokhoyan, R., Wu, P. C. & Atwater, H. A. Electro-optically tunable multifunctional metasurfaces. *ACS nano* **14**, 6912-6920 (2020).

9. Burla, M. et al. 500 GHz plasmonic Mach-Zehnder modulator enabling sub-THz microwave photonics. *Apl Photonics* **4**, 056106 (2019).

10. Haffner, C. et al. All-plasmonic Mach–Zehnder modulator enabling optical high-speed communication at the microscale. *Nature Photonics* **9**, 525-528 (2015).

11. Pai, S. et al. Experimentally realized in situ backpropagation for deep learning in photonic neural networks. *Science* **380**, 398-404 (2023).

12. Hinton, G. The forward-forward algorithm: Some preliminary investigations. *arXiv preprint arXiv:2212.13345*, (2022).

13. Hughes, T. W., Minkov, M., Shi, Y. & Fan, S. Training of photonic neural networks through in situ backpropagation and gradient measurement. *Optica* **5**, 864-871 (2018).

14. Zhou, T. et al. In situ optical backpropagation training of diffractive optical neural networks. *Photonics Research* **8**, 940-953 (2020).

15. Momeni, A., Rahmani, B., Malléjac, M., Del Hougne, P. & Fleury, R. Backpropagation-free training of deep physical neural networks. *Science* **382**, 1297-1303 (2023).

16. Brown, T. et al. Language models are few-shot learners. *Advances in neural information processing systems* **33**, 1877-1901 (2020).

17. Boyd, R. W. *Nonlinear optics*. Elsevier (2003).

18. Fisher, R. A. *Optical phase conjugation*. Academic press (2012).

19. Tietze, U. & Schenk, C. *Advanced electronic circuits*. Springer Science & Business Media (2012).

20. Wuttig, M., Bhaskaran, H. & Taubner, T. Phase-change materials for non-volatile photonic applications. *Nature photonics* **11**, 465-476 (2017).
